# Integrated multi-cohort transcriptional meta-analysis of neurodegenerative diseases

**DOI:** 10.1186/s40478-014-0093-y

**Published:** 2014-09-04

**Authors:** Matthew D Li, Terry C Burns, Alexander A Morgan, Purvesh Khatri

**Affiliations:** 1 Stanford University School of Medicine, Stanford, 94305 CA USA; 2 Department of Neurosurgery, Stanford University, Stanford, 94305 CA USA; 3 Stanford Institute for Immunity, Transplantation and Infection (ITI), Stanford Center for Biomedical Informatics Research (BMIR), Department of Medicine, Stanford University, 1265 Welch Rd., Stanford, 94305 CA USA

**Keywords:** Meta-analysis, Transcriptome, Neurodegeneration, Aging, Biomarkers, Therapeutic targets

## Abstract

**Introduction:**

Neurodegenerative diseases share common pathologic features including neuroinflammation, mitochondrial dysfunction and protein aggregation, suggesting common underlying mechanisms of neurodegeneration. We undertook a meta-analysis of public gene expression data for neurodegenerative diseases to identify a common transcriptional signature of neurodegeneration.

**Results:**

Using 1,270 post-mortem central nervous system tissue samples from 13 patient cohorts covering four neurodegenerative diseases, we identified 243 differentially expressed genes, which were similarly dysregulated in 15 additional patient cohorts of 205 samples including seven neurodegenerative diseases. This gene signature correlated with histologic disease severity. Metallothioneins featured prominently among differentially expressed genes, and functional pathway analysis identified specific convergent themes of dysregulation. MetaCore network analyses revealed various novel candidate hub genes (e.g. *STAU2)*. Genes associated with M1-polarized macrophages and reactive astrocytes were strongly enriched in the meta-analysis data. Evaluation of genes enriched in neurons revealed 70 down-regulated genes, over half not previously associated with neurodegeneration. Comparison with aging brain data (3 patient cohorts, 221 samples) revealed 53 of these to be unique to neurodegenerative disease, many of which are strong candidates to be important in neuropathogenesis (e.g. *NDN*, *NAP1L2*). ENCODE ChIP-seq analysis predicted common upstream transcriptional regulators not associated with normal aging (*REST, RBBP5, SIN3A, SP2, YY1, ZNF143*, *IKZF1*). Finally, we removed genes common to neurodegeneration from disease-specific gene signatures, revealing uniquely robust immune response and JAK-STAT signaling in amyotrophic lateral sclerosis.

**Conclusions:**

Our results implicate pervasive bioenergetic deficits, M1-type microglial activation and gliosis as unifying themes of neurodegeneration, and identify numerous novel genes associated with neurodegenerative processes.

**Electronic supplementary material:**

The online version of this article (doi:10.1186/s40478-014-0093-y) contains supplementary material, which is available to authorized users.

## Introduction

Neurodegenerative diseases promise to exert an increasingly onerous toll on society’s aging population in the coming years [1]. However, despite decades of research and hundreds of unique animal models, no therapy has yet emerged to overcome the insidious loss of neurons in neurodegenerative diseases such as Alzheimer’s disease (AD), Parkinson’s disease (PD), Huntington’s disease (HD), amyotrophic lateral sclerosis (ALS), or frontotemporal lobar dementia (FTLD). The diversity of identified contributors to neurodegeneration, including vascular pathology [2], excitotoxicity [3], oxidative stress [4], prior traumatic brain injury [5], environmental exposures [6], and genetic mutations in mitochondrial [7], RNA processing [8], proteasomal and autophagy-related genes [9], points to a multiple-hit hypothesis of neurodegeneration. In contrast to deterministic animal models wherein neurodegeneration may be induced through a single mutation and cured with a single compound [10], most human neurodegeneration occurs sporadically and may therefore reflect the cumulative effects of numerous low penetrance risk factors and stressors.

Despite the challenges posed in identifying the individual causes of neurodegeneration, common themes of mitochondrial dysfunction, protein aggregation, oxidative stress and neuroinflammation have emerged in most neurodegenerative diseases [11],[12]. Increasing numbers of transcriptome studies have addressed individual neurodegenerative diseases, including those focused on understanding regional susceptibility [13], disease progression [14], cell type-specific signals [15], and disease-specific meta-analysis [16]. However, these studies are usually limited by relatively small sample sizes and significant heterogeneity between experiments, particularly in the tissue sampled, expression analysis platform, sample procurement method, and background of the investigated patient populations. Availability of large amounts of expression profiling data in public repositories such as the NCBI Gene Expression Omnibus (GEO) and EBI ArrayExpress presents novel opportunities to carry out an integrated multi-cohort analysis of diseases, and such data has been used to identify common transcriptional signatures in cancer and infections [17],[18]. There are approximately 40 publicly available gene expression microarray studies that profiled brain tissue in neurodegenerative diseases. Collectively these studies better represent the heterogeneity of neurodegeneration observed in the real world as different research groups carried out these experiments independently using different tissue samples and microarray technologies. However, this inherent heterogeneity in public data also presents challenges in terms of how to integrate these independent studies cohesively into a single analysis. We recently proposed a meta-analysis approach that leverages the heterogeneity across different data sets to identify robust, reproducible disease gene signatures. We have successfully used this meta-analysis approach to reveal novel insights into lung cancer [19] and to predict FDA-approved drugs that can be repurposed to treat organ transplant patients [20].

No systematic multi-cohort analysis has yet evaluated transcriptional alterations that are conserved across neurodegenerative diseases. We applied our meta-analysis approach to analyze publicly available gene expression datasets of post-mortem central nervous system (CNS) tissue for AD, HD, PD, and ALS. We hypothesized that such an analysis would identify the transcriptional alterations that define neurodegeneration, regardless of the specific neurodegenerative disease. Our results identified a conserved signature of neurodegeneration, applicable even to variants of FTLD, which were not included in the original meta-analysis. We analyzed this signature with respect to normal aging brain gene expression data, cell type specificity, microglial polarization and gliosis, revealing novel insights into the neurodegenerative process. Finally, we identified patterns of gene dysregulation unique to each neurodegenerative disease relative to the others.

## Materials and methods

**Figure 1:**
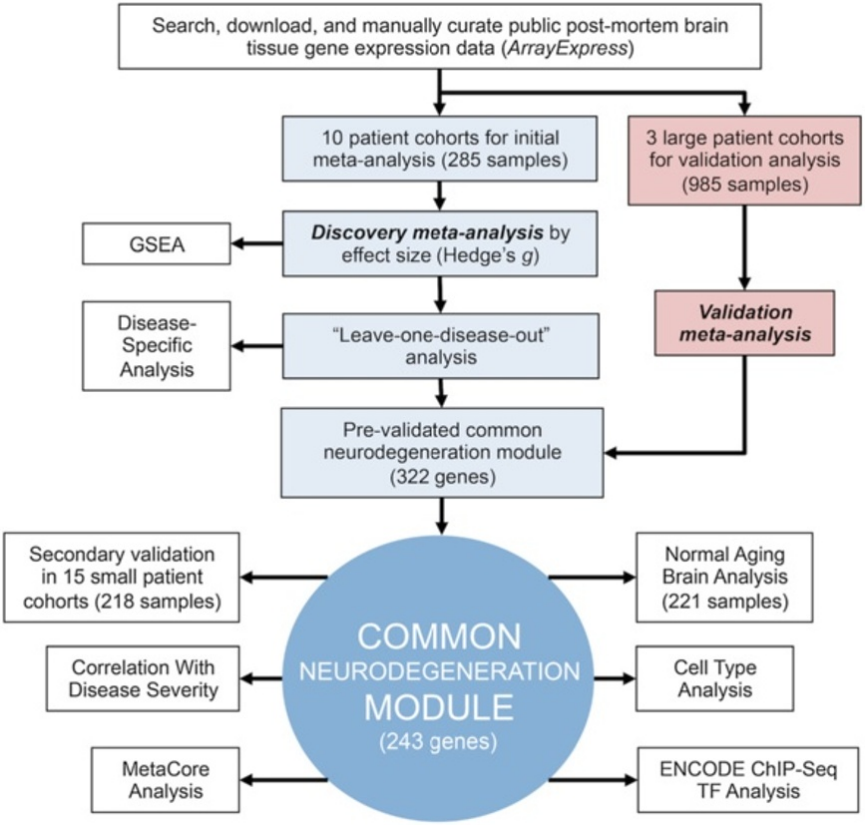
**Meta-analysis workflow schematic.** See Materials and Methods for details. GSEA, Gene Set Enrichment Analysis; TF, transcription factor.

All analyses were completed in R/Bioconductor unless otherwise noted. Heat maps were generated using the R package pheatmap [21]. The analysis workflow is shown in Figure 1.

### Data collection and pre-processing

We searched the public data repository ArrayExpress (search date: March 15, 2014) for gene expression microarray data sets from neurodegenerative disease experiments using the search terms “neurodegeneration,” “dementia”, “Alzheimer”, “Parkinson”, “Huntington”, “amyotrophic lateral sclerosis”, “frontotemporal”, “motor neurone disease”, “spinocerebellar ataxia”, “spinal muscular atrophy” and “prion”. We first identified data sets that satisfied the following criteria: (1) samples were from human post-mortem CNS tissue samples, (2) the data was originally acquired using a genome-wide gene expression microarray platform, (3) the microarray platform had reasonably accessible and clear probe-to-gene mapping annotations and (4) there were ≥5 cases and ≥5 controls total for the relevant patient cohort in each data set. We identified a total of 28 patient cohorts containing 1475 samples from 19 independent data sets that satisfied these criteria. Note that some data sets included more than one disease: we refer to each disease-specific group and its respective control group as a *patient cohort*.

Next, we divided these patient cohorts into two groups based on their sample sizes. We chose smaller patient cohorts (<100 samples) for the initial meta-analysis (*Discovery cohorts*), and we reserved larger patient cohorts (>100 samples) for validation analysis (*Validation cohorts*). For the discovery cohorts, we ensured that there were at least two patient cohorts for each disease that met our selection criteria. We then identified up to three different CNS regions affected by the disease process at the transcriptional level in each specific neurodegenerative disease, as identified in the included studies [16],[22]-[37] (e.g. AD pathology involves the entorhinal cortex, hippocampus and frontal cortex; see Table 1 for all CNS regions used). We separated the patient cohorts by these CNS regions, and we selected the largest independent cohort by sample size for each region. Thus, in the discovery cohorts, we used two to three different disease-affected brain regions for each disease. If a patient cohort contained samples from multiple CNS regions in the same individuals, we used that data set only once, selecting samples from a single CNS region. This approach ensured that every sample in the analysis came from a different individual. When possible, for data sets including multiple CNS regions, we took advantage of the opportunity to use disease-affected regions not represented in other data sets to ensure regional generalizability. Based on these criteria, we chose 10 patient cohorts containing 285 samples to include in the discovery cohorts.

**Table 1:** **Summary of public gene expression data sets used in the discovery, validation, and secondary validation data set meta-analyses**

Disease		Accession #	Study first author	Year	Citation	Cases	Controls	Total samples	Tissue source
**Discovery**	ALS	GSE4595	Lederer	2007	[22]	11	9	20	Motor cortex
	ALS	GSE26927	Durrenberger	2012	[23]	10	10	20	Cervical spinal cord
	HD	GSE3790	Hodges	2006	[24]	19	16	35	Motor cortex
	HD	GSE26927	Durrenberger	2012	[23]	10	10	20	Ventral head of caudate nucleus
	PD	GSE7621	Lesnick	2007	[25]	16	9	25	Substantia nigra
	PD	E-MTAB812	Dumitriu	2012	[26]	27	26	53	Dorsolateral prefrontal cortex
	PD	GSE20291	Zhang	2005	[27]	15	20	35	Putamen
	AD	GSE29378	Miller	2013	[28]	16	16	32	Hippocampus (CA1)
	AD	GSE36980	Hokama	2013	[29]	15	12	27	Frontal cortex
	AD	GSE26927	Durrenberger	2012	[23]	11	7	18	Entorhinal cortex
**TOTAL**						**150**	**135**	**285**	
**Validation**	HD	GSE33000	Zhu	2013	^a^	157	155	312	Dorsolateral prefrontal cortex
	AD	GSE33000	Zhu	2013	^b^	310	^c^	310	Dorsolateral prefrontal cortex
	AD	GSE15222	Webster	2009	[30]	176	187	363	Various cortical regions
**TOTAL**						**643**	**342**	**985**	
**Secondary validation**	PD	GSE26927	Durrenberger	2012	[23]	12	8	20	Substantia nigra
	PD	GSE20333	Grunblatt	2004	[31]	6	6	12	Substantia nigra
	PD	GSE20164	Hauser	2005	[32]	6	5	11	Substantia nigra
	PD	GSE20163	Zheng	2010	[16]	8	9	17	Substantia nigra
	PD	GSE8397	Moran	2006	[33]	9	7	16	Lateral substantia nigra
	PD	GSE7307	Roth	2008	^a^	5	13	18	Putamen
	PD	GSE19587	Lewandowski	2010	[34]	6	5	11	Dorsal motor nucleus of Vagus nerve
	PD	GSE20146	Zheng	2010	[16]	10	10	20	Globus pallidus interna
	AD	GSE1297	Blalock	2004	[35]	22	9	31	Hippocampus
	AD	E-MEXP2280	Bronner	2009	[36]	5	5	10	Medial temporal lobe
	PiD	E-MEXP2280	Bronner	2009	[36]	5	^d^	5	Medial temporal lobe
	PSP	E-MEXP2280	Bronner	2009	[36]	5	^d^	5	Medial temporal lobe
	FTLD (C)	E-MEXP2280	Bronner	2009	[36]	5	^d^	5	Medial temporal lobe
	FTLD-U (GRN+)	GSE13162	Chen-Plotkin	2008	[37]	6	8	14	Frontal cortex
	FTLD-U (GRN-)	GSE13162	Chen-Plotkin	2008	[37]	10	^e^	10	Frontal cortex
**TOTAL**						**120**	**85**	**205**	

See Additional file 1: Table S1 for individual sample accession numbers. ALS, amyotrophic lateral sclerosis; HD, Huntington’s disease; PD, Parkinson’s disease; AD, Alzheimer’s disease; PiD, classical Pick’s disease; PSP, progressive supranuclear palsy; FTLD, frontotemporal lobar dementia; FTLD (C), FTLD Constantinidis type C; FTLD-U, FTLD with ubiquitin- and TDP-43-positive inclusions; GRN+/–, progranulin mutation positive/negative. ^a^not published yet; ^b^published in part in [38]; ^c^same control samples as the GSE33000 HD data; ^d^same control samples as the E-MEXP-2280 PiD data; ^e^same control samples as the GSE13162 FTLD-U (GRN+) data.

For the validation cohorts, we used three patient cohorts containing 985 samples from AD and HD patients. We performed a secondary validation meta-analysis on the 15 remaining patient cohorts (205 samples) that met our inclusion criteria, which included several smaller studies of PD and AD as well as five variants of FTLD. The GEO accession numbers of the data sets used in our analysis are summarized in Table 1[16],[22]-[37].

Because the available data sets use many different microarray platforms, we downloaded the processed gene expression data for each data set, most of which were associated with peer-reviewed publications analyzing the data. Phenotypic data for each sample were also extracted where available. We log2 transformed and quantile normalized gene expression signal intensities within each data set, if not already processed as such.

### Gene expression meta-analysis

We conducted gene expression meta-analysis by combining effect sizes (standardized mean differences) as previously described in detail [20]. This approach determines a meta-effect size for each gene, which estimates the change in gene expression across all data sets given a common two-class comparison (i.e. disease vs. control). Microarray probes from each data set were mapped onto HUGO gene symbols. If a probe matched more than one gene, an additional record was added for each mapped gene. The effect size for each gene in each data set was estimated as Hedge’s adjusted *g*. If multiple probes matched to a gene, that gene’s effect size was summarized using the fixed effects inverse-variance model. Study-specific effect sizes were then combined to determine the pooled effect size and its standard error using the random effects inverse-variance technique. Nominal p-values were determined by comparing a Z-statistic (ratio of the pooled effect size to its standard error for each gene) to a standard normal distribution. The p-values were corrected for multiple hypotheses testing using Benjamini-Hochberg false discovery rate (FDR) correction [39]. In the discovery meta-analysis, genes were deemed to be significantly differentially expressed if FDR ≤ 5% and the gene was measured in all 10 patient cohorts.

### Leave-one-disease-out analysis

In order to ensure that our meta-analysis was not influenced by or biased towards a specific neurodegenerative disease, we repeated our meta-analysis four times by removing data sets corresponding to one disease at a time (e.g. in the first iteration, HD data sets were removed, and the meta-analysis was completed on the combined AD, PD, and ALS data sets). At each iteration, we identified significantly differentially expressed genes (FDR ≤ 5%). Genes that were significant, irrespective of which subset of neurodegenerative diseases were analyzed, formed the pre-validation *common neurodegeneration module* (CNM). We have previously shown the utility of the leave-one-disease-out approach in identifying a robust gene expression signature during acute rejection across different transplanted solid organs [20].

### Validation analyses

We first validated each of the genes in the CNM in the three large patient cohorts, including two AD data sets [30],[38] and a HD data set (not yet published) (Table 1). We used the meta-analysis approach described above to identify significantly differentially expressed genes across the three validation data sets (FDR ≤ 5%). Genes that were significantly differentially expressed in the same direction in both the discovery and validation analyses were considered validated. We removed the genes from the CNM that were not validated in the independent cohorts.

We further validated the CNM in 15 additional patient cohorts containing 205 samples from 10 studies of PD, AD, and variants of frontotemporal dementia (Table 1).

### Analysis of the CNM association with histologic disease severity

Some of the data sets used provided neuropathological annotations. In three data sets from the discovery cohorts, there were Braak stage and Huntington grade information for each sample [24],[28],[29]. To assess the CNM’s association with histologic disease severity, we calculated the geometric mean of the gene expression intensity for the up-regulated and down-regulated components of the CNM separately within each sample. The geometric mean of the CNM was centered and standardized across all samples in a given experiment, giving a z-score. We also calculated the difference between the up-regulated CNM z-score and the down-regulated CNM z-score in each sample. Jonckheere trend test was used to evaluate the significance of trends (two-tailed test). We generated bar plots using the R package ggplot2 [40].

### Gene ontology, pathway, and network analysis

We used Gene Set Enrichment Analysis (GSEA) [41] to identify the enrichment of pre-established gene sets across neurodegenerative diseases. We used the GSEA PreRank option to input the complete list of genes with their corresponding meta-effect sizes from the discovery meta-analysis, regardless of significance. This approach allowed us to first assess pathway enrichment without arbitrary thresholds for significance. We used the curated gene sets for Gene Ontology (GO) terms from the Broad Institute’s Molecular Signature Database (MSigDB). We set the false discovery rate q-value ≤ 0.05 as the threshold for significance. We constructed networks of overlapping significantly enriched gene sets using the EnrichmentMap plugin in the Cytoscape software [42],[43].

The MetaCore software suite (Thomson Reuters) was used to functionally analyze the CNM and generate gene networks. We set the background gene list in MetaCore to all of the genes assessed in all 10 discovery cohorts. We conducted enrichment analysis of the CNM for MetaCore’s curated pathways. We then generated a network from CNM genes using only the direct interactions between network objects. We generated additional networks using the default “analyze network” algorithm in MetaCore (50 nodes per sub-network).

The MetaCore “Interactions by Protein Functions” tool was used to identify proteins that are functionally over-connected with proteins corresponding to genes in the CNM. We opted to include protein complexes in this analysis.

### Correlation with normal aging

We investigated the correlation of each gene in the CNM with normal brain aging. We searched the EBI ArrayExpress for aging CNS microarray data sets. We identified three independent normal aging human CNS data sets (221 samples) from various tissues that had a minimum of 30 samples per experiment covering a broad age range (Table 2) [44]-[46]. For a data set that used samples from multiple CNS areas, we only used samples from the hippocampus because it has been reported to vary the least based on gender [44]. For each CNM gene in each data set, we determined Kendall’s tau coefficient between log2 transformed gene signal intensity and age using the “Kendall” R package [47]. In this package, when ties are present in the data, a normal approximation with continuity correction is made. If more than one probe existed for each gene, the geometric mean of the signal intensity of the multiple probes was used. Genes that were positively (negatively) correlated with a p-value ≤ 0.05 in ≥2 out of 3 of the normal aging CNS data sets were deemed to be significantly positively (negatively) correlated with aging.

**Table 2:** **Summary of public gene expression data from normal aging human brain studies used in analysis**

Accession #	Sample tissue source	Citation	Total samples	Age range
GSE11882	Hippocampus	[44]	43	20 to 99 years
GSE1572	Frontal Cortex	[45]	30	26 to 106 years
GSE30272	Dorsolateral Prefrontal Cortex	[46]	148^a^	20 to 78 years
**TOTAL**			**221**	

^a^data set restricted to individuals > 20 years old, lowering the sample size to 148.

We used the Database for Annotation, Visualization and Integrated Discovery (DAVID) [48] tool to assess the enrichment for GO terms in the aging correlated and non-correlated components of the CNM.

### Assessment of cell type specificity in differentially expressed genes

To evaluate whether the CNM may reflect changes in cell-type composition, we assessed the overlap between our module and genes enriched in isolated neurons, astrocytes, oligodendrocytes [49],[50], microglia or peripheral macrophages [51],[52] from normal mice, as well as genes enriched in astrocytes isolated from mice following stroke or LPS treatment [53]. While these data sets were derived from multiple experiments and could not be compared directly, they all used the same platform, the Affymetrix Mouse Genome 430 2.0 Array. This permitted us to use the Gene Expression Commons (GEXC) tool [52] to evaluate gene expression activity in these cell type specific data sets relative to 11,939 public gene array data sets, which allows for the classification of genes as “active” or “inactive”. We used the “Expression Pattern Search” function in GEXC to identify genes from the CNM that were “active” in the cell type of interest and “inactive” in all others. We then performed manual visualization of differential gene expression in GEXC to confirm and finalize the assigned cell-type enrichment category for each gene in the CNM. For genes only modestly differentially expressed between neurons, oligodendrocytes and astrocytes, we deferred to the directly compared cell type-specific gene lists from [49]. Genes not enriched in a single given cell type based on these criteria were regarded as not being cell-type specific.

### Assessment of enrichment for microglia polarization states and gliosis

We created custom gene sets from published gene lists generated from transcriptome analyses of human M1 and M2-polarized macrophages [54], microglia from the end stage of a mouse model of ALS [55], and astrocytes from mice 24 hours following treatment with lipopolysaccharide (LPS) or middle cerebral artery occlusion (MCAO) [53]. For the mouse data, we downloaded the gene lists and converted mouse gene symbols to human HUGO gene symbols prior to inputting the custom gene sets into GSEA [41]. We used the GSEA PreRank option to assess the enrichment for these custom gene sets in the complete list of discovery meta-analysis genes with their corresponding meta-effect sizes, regardless of significance.

### Identification of enriched transcription factors using ENCODE data

We used the ENCODE ChIP-Seq Significance Tool [56] to identify transcription factors enriched in the up-regulated and down-regulated components of the CNM. We used the following parameters: organism, human (hg19); regulatory element type, protein-coding genes; ID type, symbol; background regions, a list of all genes assessed across all 10 discovery data sets; analysis window center, TSS/5’ end (transcription start site); upstream and downstream window size relative to TSS, 500 bp; and cell lines, all. We repeated this analysis for genes positively and negatively correlated with aging and for published gene lists where indicated.

### Identification of unique disease-specific patterns of gene expression changes

To identify patterns in gene expression changes that are unique to each of the neurodegenerative diseases evaluated, for each specific disease, we repeated the aforementioned gene expression meta-analysis on the disease by itself, as well as separately on the other three diseases together. We then removed genes from the individual disease meta-analysis output gene list that were significantly differentially expressed from the three-disease meta-analysis (FDR ≤ 0.05), thereby omitting common differentially expressed genes in neurodegeneration from disease-specific gene lists. We restricted the gene lists to genes that were measured in all 10 data sets. The resulting individual disease meta-analysis gene list was then input into GSEA PreRank for assessment of GO term enrichment as described earlier.

## Results

### Meta-analysis identifies a common gene signature of neurodegeneration

For our discovery meta-analysis of neurodegenerative diseases, we collected microarray data sets containing 10 independent patient cohorts that profiled human post-mortem CNS tissues in 285 samples (150 cases, 135 controls) (Table 1, Additional file 1: Table S1) [22]-[29]. These samples were obtained from various cortical regions, hippocampus, basal ganglia, and spinal cord in four neurodegenerative diseases (AD, PD, HD, and ALS). These experiments used seven different gene expression microarray platforms. As some data sets do not provide raw data and optimal microarray pre-processing techniques differ across platforms, we downloaded processed signal intensities, and checked that all data were log2 transformed and quantile normalized across all samples in the specific experiment. If not, we log2 transformed and quantile normalized the data. We used disease phenotypes as defined in the original publications for disease versus control tissue comparisons. The included studies also generally showed an effort to age-match cases and controls.

To identify the most robust and consistently differentially expressed genes across neurodegenerative diseases, we used a gene expression meta-analysis approach [20]. Briefly, this approach combines the effect sizes, calculated as Hedges’ adjusted *g*, for each gene from each data set to estimate a standardized mean difference in gene expression (see Materials and Methods for details). For a gene to be considered differentially expressed in the meta-analysis, we required it to be measured in all 10 patient cohorts and for its effect size to have a significant false discovery rate (FDR ≤ 5%). This analysis yielded lists of 3,078 and 3,565 significantly up-regulated and down-regulated genes, respectively (Additional file 1: Table S2).

However, because of the heterogeneity in effect sizes, it is possible that some of the genes may be differentially expressed in one or more neurodegenerative diseases, but not all. Because our goal is to identify a set of common genes that are differentially expressed in the same direction across all neurodegenerative diseases, we carried out “leave-one-disease-out” analysis. In this analysis, we repeated the meta-analysis four additional times, each time removing patient cohorts corresponding to one disease prior to analysis of the remaining patient cohorts for the other three diseases. Genes that remained significantly differentially expressed (FDR ≤ 5%) in all four iterations of the “leave-one-disease-out” analysis were considered to represent the common genes dysregulated across neurodegenerative diseases. We identified 322 such consistently differentially expressed genes (95 up-regulated, 227 down-regulated) irrespective of which subset of neurodegenerative diseases were analyzed (Additional file 1: Table S3). It is possible that there may still be significant heterogeneity in the effect sizes of these genes between different neurodegenerative diseases; however, this heterogeneity may indicate that the same pathway is expressed at different levels between neurodegenerative diseases, but in the same direction. Therefore, we did not further consider this heterogeneity for the 322 genes.

**Figure 2:**
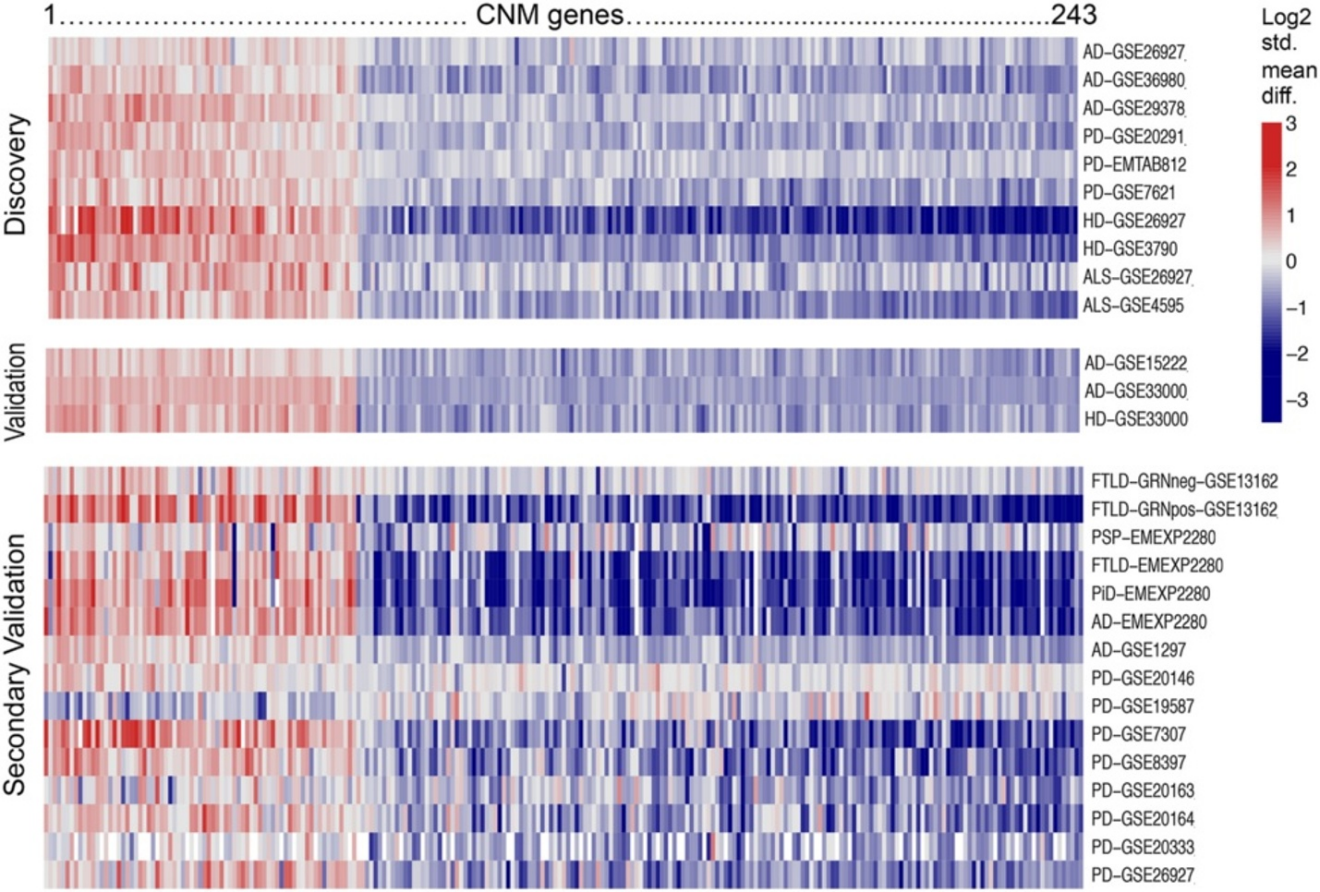
**Meta-analysis and leave-one-disease-out analysis reveal common differentially expressed genes across neurodegenerative diseases.** Heat map shows consistent differential expression in the discovery, validation, and secondary validation data sets. Columns denote CNM genes ranked from highest to lowest standardized mean difference (Hedges’ *g* in log2 scale), from left to right. Rows denote data sets used in each stage of meta-analysis. Heat map colors indicate Hedges’ *g* in log2 scale. Refer to Table 1 for data set information. ALS, amyotrophic lateral sclerosis; HD, Huntington’s disease; PD, Parkinson’s disease; AD, Alzheimer’s disease; PiD, classical Pick’s disease; FTLD, frontotemporal lobar dementia (Constantinidis type C); PSP, progressive supranuclear palsy; FTLD-GRNpos, frontotemporal lobar dementia with ubiquitin- and TDP-43-positive inclusions, progranulin mutation positive; FTLD-GRNneg, frontotemporal lobar dementia with ubiquitin- and TDP-43-positive inclusions, progranulin mutation negative.

Next, we validated these genes in three additional patient cohorts of neurodegenerative disease consisting of 985 samples (643 cases, 342 controls) (Table 1, Additional file 1: Table S1) [30],[38]. These data sets profiled human post-mortem CNS tissue samples from patients with AD or HD. Large validation data sets were not publicly available for PD and ALS. We found 73/95 (76.8%) up-regulated genes and 170/227 (74.9%) down-regulated genes (total of 243 genes) were also significantly differentially expressed in the validation cohorts (Figure 2 and Additional file 2: Figure S1). Henceforth, these 243 validated genes are referred to as a *common neurodegeneration module* (CNM) (Table 3 and Additional file 1: Table S4).

**Table 3:** **Common neurodegeneration module (CNM) genes**

Up-regulated		Down-regulated				
NUPR1	**CXCR4**	RGS4	**PSMC3**	OXCT1	**ATP5C1**	**DLG3**
**NXT1**	**ELF1**	DCLK1	PDHB	**ATP6V1A**	**PPEF1**	**RPL15**
MT2A	**CEBPB**	**PCP4**	**PLCB1**	**HARS**	**RTN3**	**COQ7**
ITPKB	**RAB7L1**	**INA**	**GABRA1**	**RFK**	**TUBG2**	**ACSL6**
MT1X	**THOC1**	ATP6V1G2	**HMP19**	**NEDD8**	**AP3B2**	**RFC3**
MT1H	**IFITM3**	**TAC1**	TAGLN3	**RNMT**	**CEP170B**	**COPS8**
AEBP1	**IFITM2**	**REEP1**	**NDN**	**CACNB1**	**FIS1**	**API5**
MT1F	SLCO4A1	**SCG3**	**G3BP2**	LANCL1	**MKKS**	**GLYR1**
HSPB1	**TIPARP**	CHGB	**UBE2N**	**TCEAL4**	ACP1	**CLASP2**
**TNFRSF1A**	LRP10	**SST**	CDK5	**NLK**	TIMM17A	**LRRC8B**
FAM107A	NPC2	**PCSK1**	**RPA3**	**RTCA**	**PSMD1**	
NFKBIA	**TREM2**	**HIGD1A**	**KIFAP3**	**PPP3CB**	CHN1	
**BCL6**	**IFI30**	**MOAP1**	**PEX11B**	**RNF11**	**RAB3B**	
**KCNE4**	**EFNA1**	PTS	**SLC30A9**	**BBS7**	**MICAL2**	
**ACP5**	CASP6	**GNG3**	**SLC4A1AP**	**LRPPRC**	NRXN1	
**FSTL1**	**ITPKC**	MLLT11	STMN2	**NDUFAB1**	**PGRMC1**	
CHSY1	**CD37**	ATXN10	**PFDN1**	GABRG3	**DYNC1LI1**	
PTBP1	**YBX1**	**PIN1**	**NRIP3**	**PTPRN2**	**TOR1A**	
HSPB8	**LHFPL2**	**DYNC1I1**	**CDK5R2**	TXNDC9	**ACLY**	
MSN	**YBX3**	GOT1	**ATP6V0E2**	TUBA4A	PDE2A	
**CCL2**	**TMBIM1**	**CNIH3**	MAGED1	**STXBP1**	**IMMT**	
SERPINA3	**CTBP2**	**TM7SF2**	FAM188A	**GHITM**	**ASMTL**	
**PHF10**	CFI	RAP1GDS1	**ITFG1**	RAB6A	**NAP1L2**	
TNIP2	**DRAM1**	**ATP5B**	HLF	**BPGM**	**STAU2**	
PALLD	**VAMP8**	SCG5	**TBC1D9**	**ALDH1A3**	SERINC3	
**BAG3**	CDKN1A	TCEB1	NDUFA9	**CALM1**	**FEN1**	
**MID1IP1**	**MS4A6A**	**COX8A**	TMEFF1	**SERPINI1**	CXADR	
**EMP3**	**RBBP6**	FGF12	**SNX4**	**FIBP**	**ARHGAP44**	
**CLIC1**	**CSF1**	**TSPYL5**	**DIRAS2**	ATP5A1	**EIF4H**	
**INHBB**	TGM2	**FAM216A**	**MRPS33**	**PEX19**	CD200	
**DDAH2**	**SLA**	**SYT1**	**OAT**	**LARGE**	**PTP4A1**	
**HMOX1**	**TRAF3IP2**	**COPS3**	LANCL2	CYCS	RTN1	
**MTHFD2**	**FBLN1**	**POP4**	**ISCA1**	**GRIK1**	MAGI1	
**SYNM**		**NEFL**	**PAK6**	**ARL4D**	**KHDRBS1**	
ERBB2IP		**B4GALT6**	**MTCH2**	**PPP1R2**	**CNTNAP2**	
**CMTM6**		**NDRG3**	**CAP2**	**MPC2**	**TCEA2**	
**PIM1**		**NBEA**	**GABRD**	**SLC1A1**	**TNFRSF21**	
**BTG1**		TASP1	**ENO2**	C11orf24	**SNAPC5**	
**CTDSP2**		**DLD**	SAMM50	**AP3M2**	**ELAVL4**	
**TNFRSF12A**		**ARHGEF9**	FGF13	**PJA2**	**MEAF6**	

Genes are listed from largest absolute meta-effect size to smallest, top-to-bottom, left-to-right. Genes NOT correlated with age shown in bold. See Additional file 1: Table S4 for details.

Finally, we extended our analysis to include data sets and neurodegenerative diseases that were not part of the discovery or validation cohorts in order to test the generalizability of the CNM. This secondary validation included 205 samples from 15 patient cohorts from 10 independent experiments, including PD, AD, and five variants of FTLD (Table 1, Additional file 1: Table S1). Restricting our analysis to the 243 CNM genes, 42/72 (65.3%) and 156/170 (91.7%) of the up- and down-regulated CNM genes were differentially expressed in this secondary validation meta-analysis (because some data sets did not assess all 243 CNM genes, we only required the gene to be assessed in half the experiments to be included in the analysis, but one of the 73 up-regulated CNM genes did not meet this criteria). Since these data sets were small and inherently noisy, we did not further alter our CNM gene list based on the results of this secondary validation meta-analysis. Nevertheless, visual inspection of a heat map of the CNM genes (Figure 2 and Additional file 2: Figure S2) show that the CNM pattern of expression is generally highly consistent between the discovery, validation and secondary validation meta-analyses, further supporting the generalizability of the CNM to neurodegenerative diseases.

Two PD data sets (GSE19587 and GSE20146) in the secondary validation analysis did not show the CNM pattern of expression. In GSE19587, tissue was sampled from the dorsal motor nucleus of the Vagus nerve [34], which showed uniquely decreased cerebral blood volume in PD on MRI relative to other brainstem regions. The impact of vascular perfusion on gene expression in neurodegeneration requires further evaluation. GSE20146 used samples from the globus pallidus interna [57] a region not typically associated with the neurodegenerative aspect of PD.

**Figure 3:**
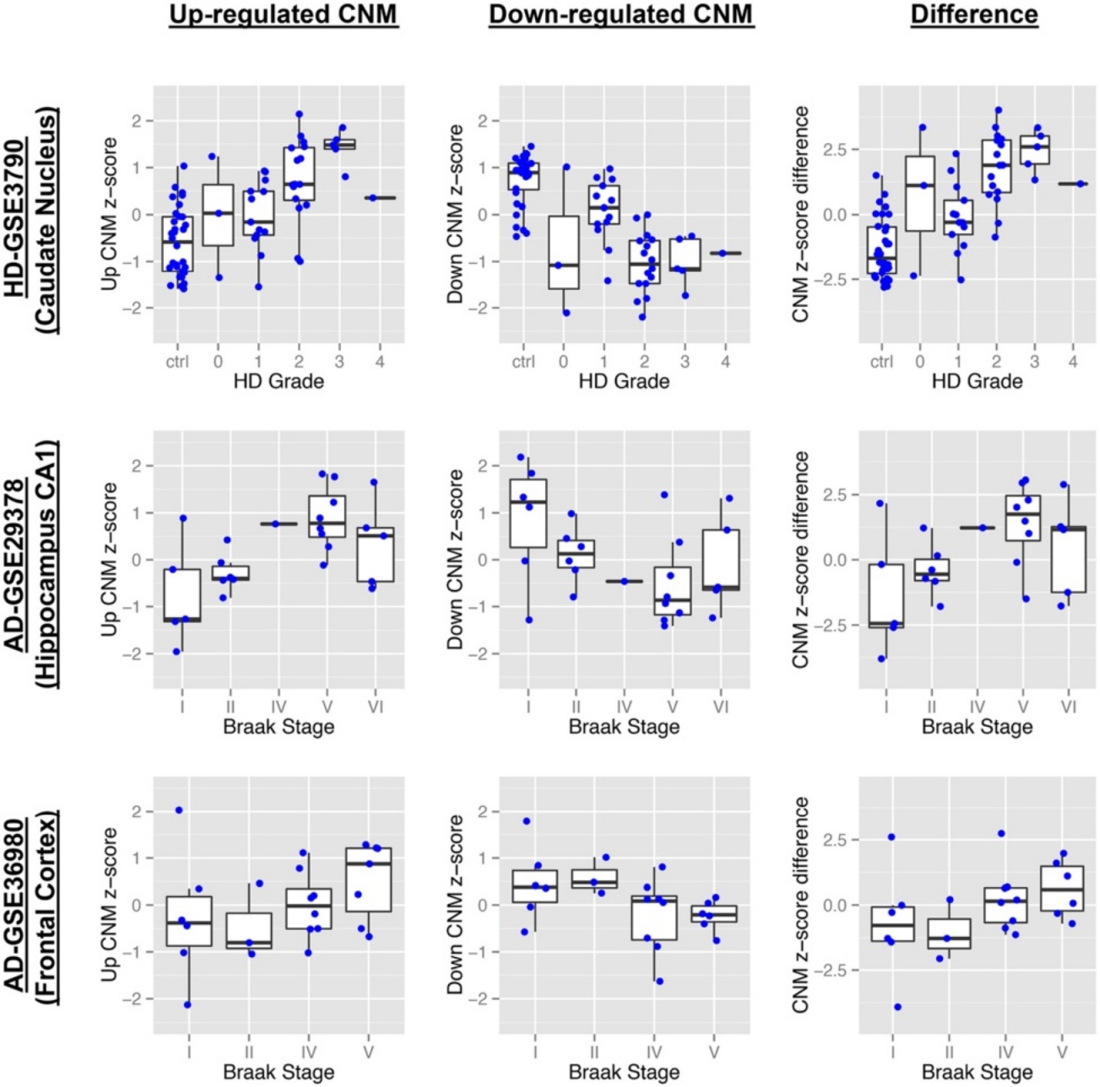
**CNM significantly associates with histologic disease severity.** Boxplots of the CNM z-score (standardized geometric mean of the up-regulated or down-regulated CNM) and the difference between the up- and down-regulated z-scores for samples in each disease neuropathology category in three independent data sets (GSE3790, GSE29378, GSE36980). Blue dots correspond to individual samples. The up-regulated CNM trends upward with increasing disease severity, while the down-regulated CNM trends downwards with increasing disease severity. The difference between the z-scores increases with disease progression. Jonckheere's trend test shows significant association (two-tailed p ≤ 0.05) in 8 out of 9 plots (left to right, HD-GSE3790: p = 0.00356, p = 0.00003, p = 0.00022; AD-GSE29378: p = 0.00758, p = 0.04707, p = 0.01460; AD-GSE36980: p = 0.07324, p = 0.00874, p = 0.01815). HD, Huntington’s disease; AD, Alzheimer’s disease.

In addition, we assessed the association of the CNM with histologic disease severity in individual patient samples. Three of the discovery cohorts categorized patients based upon histologic criteria of disease severity, including “HD grade” [24] and AD Braak stage [28],[29]. It should be noted that disease severity was not considered during meta-analysis, and every sample was classified as either “control” or “case.” We calculated the geometric mean of up-regulated and down-regulated CNM genes separately for each sample, as well as the difference. We found that the geometric mean of the up-regulated CNM genes increases with disease severity, while that of the down-regulated CNM genes decreases with disease severity (Figure 3). Furthermore, the difference in each sample between the geometric mean of the upregulated CNM genes and down-regulated CNM genes increases with disease severity. This trend was statistically significant (two-sided p < 0.05, Jonckheere’s trend test) in five of the six cases where the up-regulated and down-regulated components were analyzed separately and in all three cases when the difference was analyzed. In summary, the CNM represents a shared core signature of neurodegeneration and is associated with disease severity.

### Meta-analysis highlights common mechanisms of neurodegenerative diseases

**Figure 4:**
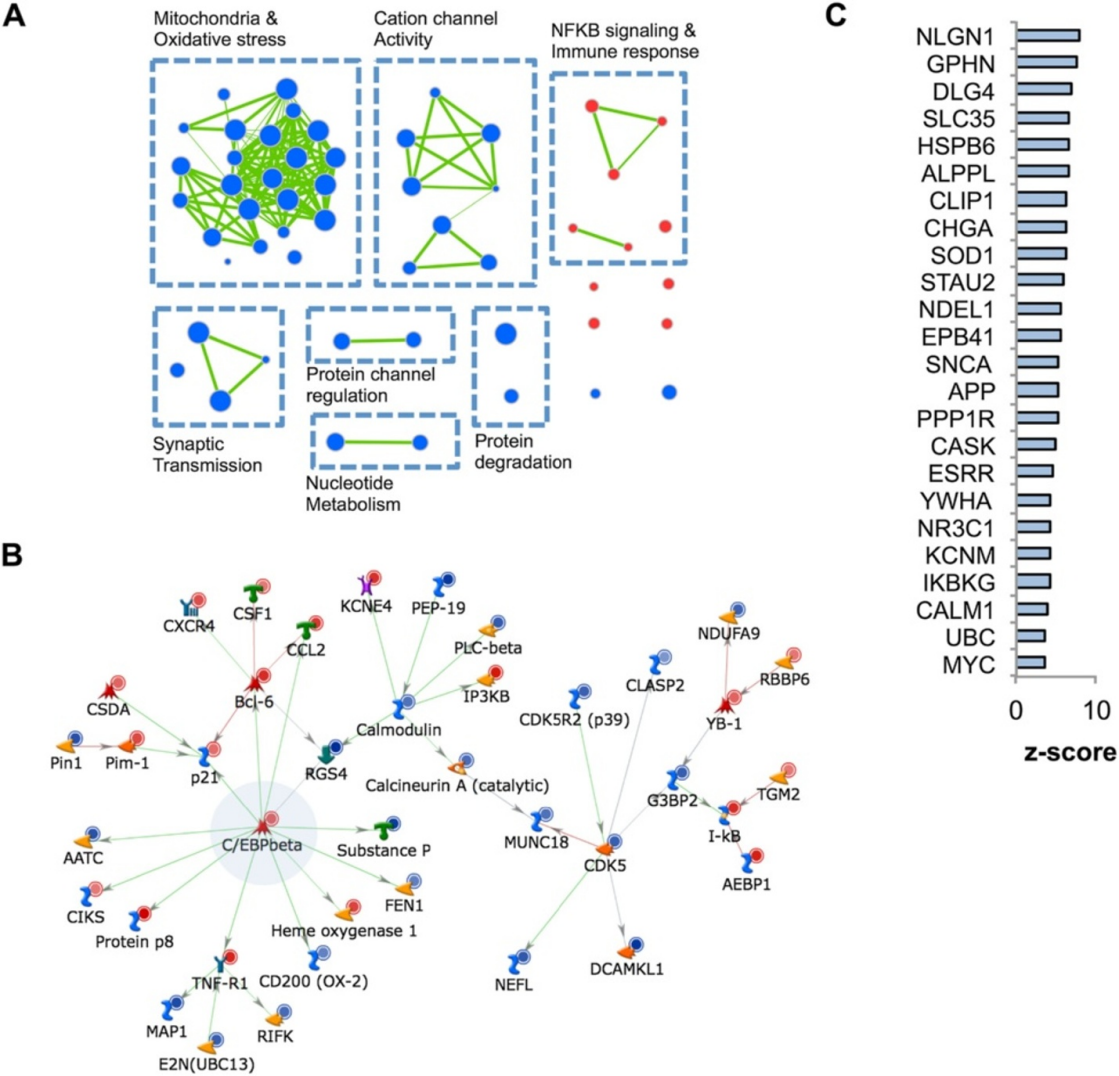
**Network and pathway analyses reveal common pathways and hubs in neurodegeneration. (A)** EnrichmentMap [42] network for overlapping enriched Gene Ontology gene sets identified by GSEA. Each node represents a significantly enriched gene set (FDR q-value ≤ 0.05), and more significant nodes are proportionally larger. Red nodes denote gene sets enriched in neurodegenerative disease tissue, while blue nodes denote those enriched in control tissue. Green lines appear between any gene sets with > 50% overlap, and are proportionally thicker given greater overlap. See Additional file 2: Figure S3 for full annotations of nodes. **(B and C)** MetaCore analyses generated, inputting all 243 CNM genes. **(B)** Network generated using only direct interactions between CNM genes. Smaller red and blue circles denote up-regulated and down-regulated genes respectively. Refer to MetaCore website for detailed network symbol legend. **(C)** MetaCore “Interactions by Protein Function” analysis identification of proteins, not necessarily within the CNM or differentially expressed at all, that are highly functionally connected with proteins corresponding to genes in the CNM. Z-score, standardized connectivity ratio (higher ratios denote greater connectivity).

We hypothesized that the results of our meta-analysis would enable us to identify conserved pathways dysregulated across neurodegenerative diseases. We used Gene Set Enrichment Analysis (GSEA) [41] to evaluate the enrichment of Gene Ontology (GO) terms in the complete ranked gene list from the discovery meta-analysis, prior to “leave-one-disease-out” analysis and validation analysis (Figure 1). Using the GSEA PreRank option and the discovery meta-analysis gene list, we found that 10 and 48 GO terms were significantly enriched (FDR ≤ 5%) in neurodegeneration and normal control tissue, respectively (Additional file 1: Table S5). We further generated networks connecting overlapping GO gene sets to aid in the interpretation of these results (Figure 4A and Additional file 2: Figure S3). We found that gene sets enriched in neurodegeneration relative to control tissue formed clusters relating to NFκB signaling, immune response and cytokine binding, whereas gene sets enriched in control tissue relative to neurodegeneration included clusters relating to mitochondrial and oxidative metabolism, cation channel activity, synaptic transmission, protein channel regulation, and nucleotide metabolism. Although not clustered together, the proteasome complex and ubiquitin cycle, which are both related to protein degradation, were both enriched in control tissue. Collectively, these findings are consistent with established literature regarding common pathways in neurodegenerative diseases, including chronic neuroinflammation, oxidative stress, mitochondrial dysfunction, altered synaptic transmission, and disrupted protein degradation [12].

### Network analyses reveal shared pathways and hub genes in neurodegeneration

To gain insight into the functional characteristics of the CNM specifically, we used MetaCore, an integrated functional analysis tool based on a manually curated database of published molecular biology data. Enrichment analysis in MetaCore for disease biomarkers, process networks, and pathway maps largely reiterated what we found in the Gene Ontology analysis, with the additional identification of gene sets related to altered cell adhesion, cytoskeletal changes, and endocrine signaling (Additional file 1: Table S6).

We then used MetaCore to generate a network, which was restricted to direct interactions between the protein products of input genes to conservatively avoid potentially spurious interactions. From the 243 CNM genes, a network of 43 directly connected proteins was identified (Figure 4B) centered on the hub gene *CEBPB. CEBPB* (CCAAT-enhancer binding protein beta), which is up-regulated in the CNM (Additional file 2: Figure S4), is a transcription factor known to be involved in regulating inflammatory responses. It has recently been shown to be up-regulated in AD and ALS microglia [58],[59]. *CEBPB* is also enriched in astrocytes, relative to neurons and oligodendrocytes [49], and has been found to be up-regulated in reactive astrocytes responding to stroke or LPS [53]. Additional hub proteins included *CDK5*, *CALM1* (part of the calmodulin protein complex), and *BCL6*. While aberrant *CDK5* and calmodulin activity have been associated with neurodegenerative diseases through tau hyperphosphorylation and calcium signaling respectively [57],[60], *BCL6* has not been previously associated with neurodegeneration. However, *BCL6* does play a role in inflammatory signaling in macrophages [61]. Three secreted proteins also appear in this network: *CSF1*, *CCL2*, and Substance P—proteins all associated with inflammatory signaling [62]-[64]. In summary, the CNM direct interaction network reveals a common network of inflammation-related protein interactions underlying neurodegenerative disease.

Removing the direct interaction restriction from the MetaCore network-building algorithm, we built additional networks using the default “analyze network” algorithm, which generates a comprehensive network of interactions based on CNM genes prior to fragmenting it into smaller, more manageable sub-networks. This analysis yielded 28 sub-networks. The top sub-network (p = 3.41 × 10^–20^) contained 19 CNM genes, and was centered on the *SP1* transcription factor (Additional file 2: Figure S5A). Although *SP1* is not a CNM gene, it was significantly up-regulated in the discovery meta-analysis list (FDR = 0.001) (Additional file 2: Figure S4). Moreover, it is elevated in AD [65], responds to oxidative stress [66], regulates expression of *APP* and tau [67], and is a proposed hub gene common to both AD and PD pathogenesis [12]. Other hub proteins in this network include *GRP78*, *NFKBIA*, *ATM*, and *YB-1*. The network is also enriched for Gene Ontology terms related to apoptosis and contains a MetaCore canonical pathway pertaining to heat shock protein and proteasome signaling that includes the proteins Parkin and Huntingtin (Additional file 2: Figure S5A). The second sub-network (p = 4.37 × 10^–12^) is centered on *NR3C1* (GCR-alpha), the glucocorticoid receptor (Additional file 2: Figure S5B). Although not differentially expressed in our meta-analysis (Additional file 2: Figure S4), GCR-alpha signaling has been implicated in neuroinflammation, particularly in relation to stress [68]. The third sub-network (p = 3.40 × 10^–12^) is centered on c-Myc (Additional file 2: Figure S5C), and appears to be related to ephrin signaling, which is implicated in aberrant synaptic function [69]. Our MetaCore network analysis identified additional common core networks of genes dysregulated across neurodegenerative diseases (Additional file 1: Table S7).

### Novel common neurodegenerative hub proteins

Next, we used the MetaCore “Interactions by Protein Function” analysis tool to identify proteins, not necessarily within the CNM or differentially expressed at all, that are highly functionally connected with proteins corresponding to genes in the CNM. This analysis allows for the identification of hub proteins that may not be dysregulated at the gene expression level, but are influencing the CNM, possibly through altered protein translation, post-translational modification or molecular interactions. We identified 24 candidate hub proteins (Figure 4C and Additional file 1: Table S8). Among these hub proteins are many that have a well-established role in neurodegeneration. *SOD1*, *SNCA*, and *APP* are central to current hypotheses around ALS, PD, and AD pathogenesis respectively [70]. As such, the hub proteins identified here may represent different disease pathologies that converge on the CNM. In addition, the top three most highly connected genes *NLGN1*, *GPHN*, and *DLG4*, as well as *PPP1R9B*, are all associated with synaptic function [71]. Not surprisingly, many other identified hub proteins have known associations with aspects of neurodegeneration. *NR3C1*, the glucocorticoid receptor, is associated with elevated stress signaling in neurodegeneration. *IKBKG* is a part of the NFκB cascade, which is associated with neuroinflammation [72]. Ubiquitin is central in protein degradation [73]. c-Myc and dysregulated cell cycling are associated with AD [74]. 14-3-3 beta/alpha is associated with Creutzfeldt-Jakob disease. *CASK* and calmodulin are associated with dysregulated calcium signaling in neurodegeneration [60]. Chromogranin A is a pro-inflammatory peptide implicated in AD and ALS [75].

In addition to providing further evidence supporting the role of these proteins in neurodegenerative processes, our analysis identified 9 hub proteins (gene symbols in parentheses if different from protein) that have not previously been implicated in neurodegenerative disease. C2orf18 (*SLC35F6*) is a protein localized to mitochondrial that is involved in apoptosis [76]. HSP20 (*HSPB6*) is a heat shock protein that may be involved in excitoxicity [77]. PLAP-like (*ALPPL2*), a germ cell alkaline phosphatase, is aberrantly expressed in seminoma [78]. CLIP170 (*CLIP1*) regulates microtubule dynamics [79]. *STAU2* is a hub gene involved in neuronal RNA transport [80] and is also down-regulated in the CNM (FDR = 7.83×10^–5^). NUDEL (*NDEL1*) is a neurodevelopment protein involved in assembly, transport and neuronal integrity [81]. *EPB41*, also known as protein 4.1R, is a part of the red cell membrane cytoskeletal network, but has been implicated in post-synaptic molecule organization [82]. ERR3 (*ESRRG*) is a nuclear estrogen receptor-related protein highly expressed in the brain [83]. MaxiK alpha subunit (*KCNMA1*) is associated with synaptic transmission [84]. These novel hub proteins may serve as candidate genes for further investigation into disease mechanisms and the development of novel therapies for neurodegenerative diseases.

### Characterizing the association of CNM genes with normal aging

Aging is an important risk factor for neurodegenerative diseases and is associated with altered microglial activity [85], synaptic plasticity [86] and a component of “normal” cognitive decline [87]. However, normal healthy aging does not involve the severe progressive loss of function observed across neurodegenerative diseases. It is known that aging is associated with increased inflammation and oxidative stress [85], but the healthy brain has adaptive strategies to maintain “normal” function in spite of the normal stresses of aging. This relentless destructive process is only observed in neurodegenerative diseases. Therefore, we hypothesized that the CNM genes that are down-regulated with neurodegeneration, but not with aging may be particularly critical to maintenance of the “healthy aging” process. Conversely, the CNM genes up-regulated specifically in neurodegeneration, but not in normal aging, may be specific drivers of progressive neurodegeneration and could be biomarkers of the degenerative process.

We identified and analyzed three independent post-mortem human microarray data sets investigating the normal aging cortex from age 20 to 106 years (Table 2). We removed samples from patients younger than 20 years of age to avoid developmental changes in gene expression. These data sets included 221 independent samples from the hippocampus, frontal cortex, and dorsolateral prefrontal cortex, all areas associated with changes in aging [44]-[46]. Heat map visualization of these data sets suggests that some genes in the CNM are correlated with aging, and these changes may be the largest in the hippocampus (Additional file 2: Figure S6).

For each gene in the CNM, we determined the non-parametric Kendall rank correlation coefficient (τ) between the gene’s log2 transformed signal intensity and age, and we calculated the two-tailed p-value for each coefficient. Genes that were significantly correlated with age (p ≤ 0.05) in the same direction in at least two out of the three independent data sets were considered correlated with age. Genes that did not meet this criterion were considered to be unchanged in aging. Among all genes evaluated in the discovery meta-analysis, 545 were positively correlated with aging, while 499 were negatively correlated with aging (Additional file 1: Table S9). Of these, we identified 126 genes that were down in the CNM and unchanged in aging (i.e., candidate genes required to prevent neurodegeneration), 48 genes that were up in the CNM and unchanged in aging (candidate neurodegeneration biomarkers), 25 genes that were up in both the CNM and aging, and 44 genes that were down in both the CNM and aging (Table 3 and Additional file 2: Figure’S6 and Additional file 1: Table S10 and Additional file 1: Table S11). No genes were detected that were up in the CNM and down in aging or down in the CNM and up in aging. The overlap between CNM genes and genes correlated with aging was highly significant (p < 2.2 × 10^–16^, Fisher’s exact test).

We used the DAVID [48] bioinformatics tool to assess the functional enrichment of the these aging-related subgroups of CNM genes with GO terms. None of the groups were significantly enriched for any terms (Benjamini-Hochberg corrected p-value ≤ 0.05), except for the GO cellular component term “mitochondrion” for the CNM genes that were down-regulated in neurodegeneration and unchanged in aging, which suggests that impaired mitochondrial function might be the most consistent specific feature of neurodegeneration, when compared to aging. This finding is consistent with mitochondria-related gene sets being the most significantly dysregulated process in our GSEA (Figure 4A and Additional file 1: Table S4).

### Assessment of CNS tissue composition and cell type-specific changes

**Figure 5:**
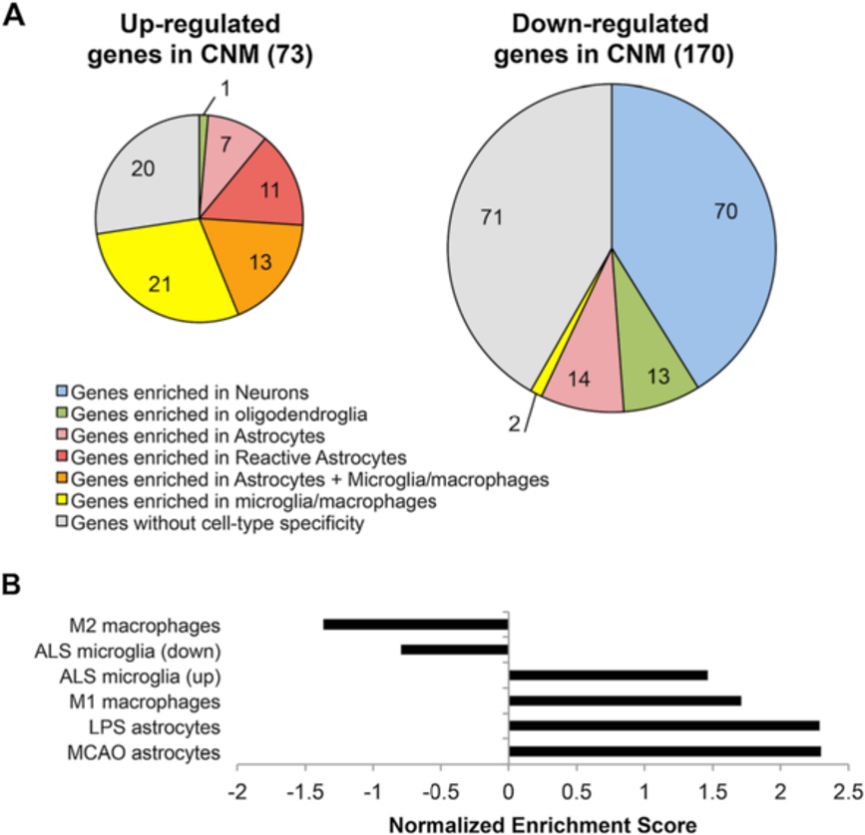
**Cell type and activation state analysis of CNM genes. (A)** Cell type-specificity of the CNM genes. CNM genes were categorized by cell type enrichment based on analysis of public data (see Materials and Methods). The distribution of genes in the categories is shown for CNM genes up-regulated (left) and down-regulated (right). **(B)** Assessment of microglia/monocyte activation and reactive astrocyte states in the discovery meta-analysis gene list. GSEA for custom gene sets for glial cell polarization states from isolated cell transcriptome analyses in the literature. Positive normalized enrichment scores indicate enrichment in neurodegeneration, while negative score indicate enrichment in control tissue. All enrichments are significant (p < 0.005), except for ALS microglia (down). ALS (up or down), amyotrophic lateral sclerosis, up-regulated or down-regulated genes (mouse model); LPS, lipopolysaccharide treated mouse, up-regulated genes; MCAO, middle cerebral artery occlusion mouse, up-regulated genes.

Because neurodegenerative diseases involve the loss of neurons, the proportion of CNS cells in a CNS tissue sample may change. As such, genes up-regulated in the CNM may reflect increased glial cell density, while genes that are down-regulated in the CNM may reflect decreased neuronal density. To test this hypothesis, we determined whether or not each gene in the CNM demonstrated a cell type-specific expression pattern based on public data sets for purified cell types, including neurons, astrocytes, reactive astrocytes, oligodendrocytes, and microglia/macrophages [49],[50],[52],[53] (Additional file 1: Table S12). We used the Gene Expression Commons to allow for comparison of gene expression between these data sets (see Materials and Methods for details) [52]. Although some astrocyte-associated genes were present in both the up and down-regulated components of the CNM, the up-regulated component of the CNM was comprised predominantly of genes enriched in reactive astrocytes, monocytes, or both—groups that were largely absent from the down-regulated portion of the CNM. Conversely, 70 of the 170 down-regulated CNM genes were enriched in neurons, whereas no neuron-specific genes were present in the up-regulated portion of the CNM (Figure 5A). These results suggest that the decrease in expression of the neuron-specific CNM genes in part could be either due to reduction in neuronal cell density in neurodegenerative disease or due to decrease in expression in neurons without any change in neuronal cell density.

Furthermore, we found that approximately half of the neuron-enriched genes have never previously been associated with neurodegeneration, despite in many cases having a variety of potentially important roles, including in neural development (Additional file 1: Table S12).

### Enrichment for activated microglial and reactive astrocyte states

Microglial activation, monocyte infiltration and gliosis are common features of neurodegenerative disease. To date, no human transcriptome data are available for microglia or reactive astrocytes from neurodegenerative disease. In order to gain insights into the transcriptional contributions of microglia/macrophages and gliosis to neurodegenerative disease, we performed GSEA [41] using custom gene sets for various defined populations of microglia/monocytes and astrocytes (Figure 5B) [53]-[55]. We observed significant enrichment in the complete discovery meta-analysis ranked gene list for genes up-regulated in mouse astrocytes responding to stroke or LPS, relative to controls (p < 0.001), as well as for genes up-regulated in human M1 polarized, relative to M2 polarized macrophages (p < 0.001). We also observed enrichment for genes up-regulated in microglia isolated from a mouse SOD1 model of ALS (p < 0.001). Of note, the discovery meta-analysis gene list was depleted (i.e. normal controls were enriched) of genes differentially expressed in human M2 macrophages (p = 0.005).

### Transcription factors associated with the CNM versus normal aging

**Figure 6:**
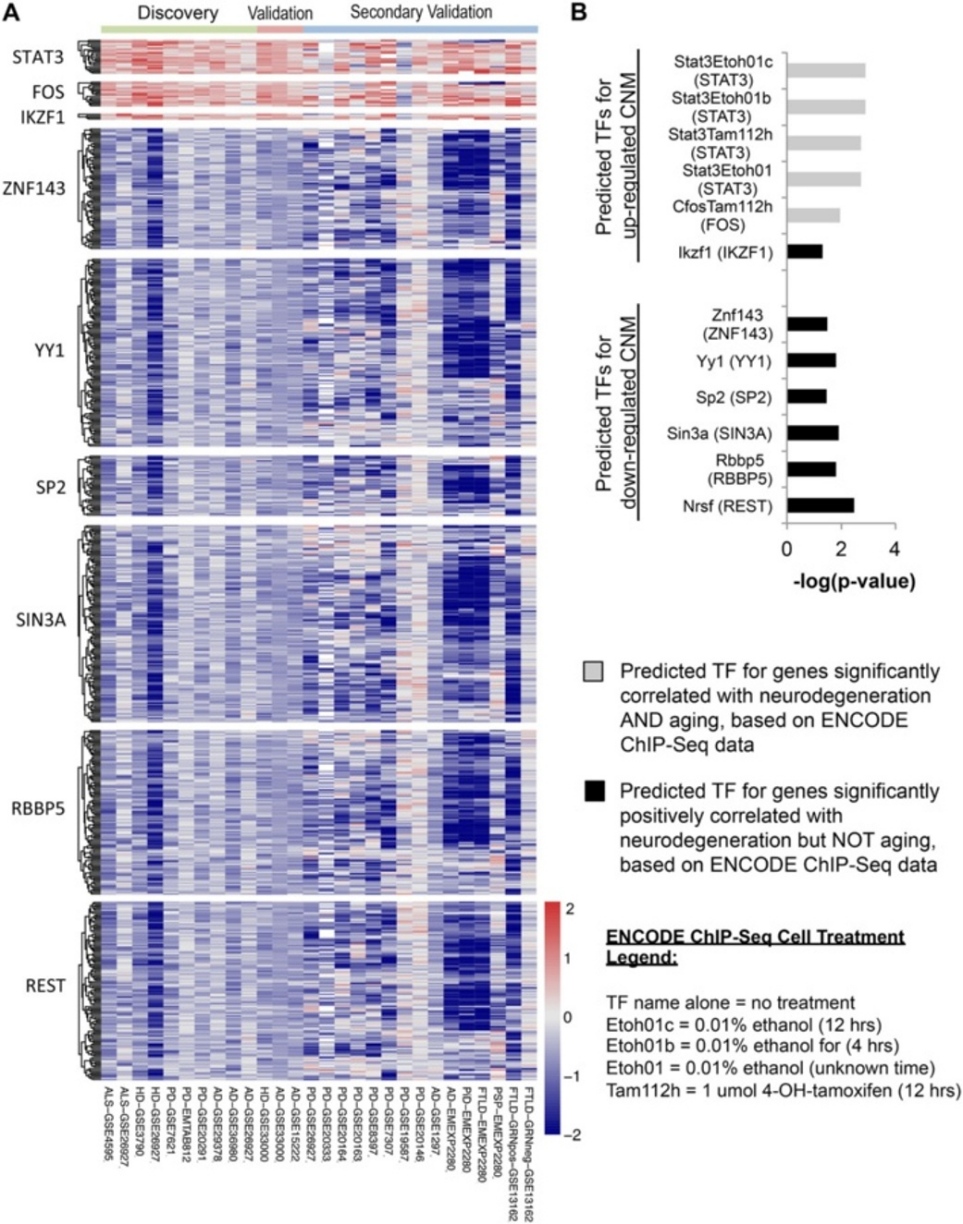
**ENCODE ChIP-seq significance analysis identifies transcription factors upstream of CNM genes. (A)** Heat map shows genes in CNM bound by transcription factors across discovery, validation, and secondary validation analyses. Heat map colors correspond to log2 standardized mean difference (Hedges’ *g*). Up and down-regulated CNM were analyzed separately. **(B)** Bar plot shows –log(q-value) for predicted transcription factors. All shown are significant (q < 0.05). Refer to Table 1 for data set information. ALS, amyotrophic lateral sclerosis; HD, Huntington’s disease; PD, Parkinson’s disease; AD, Alzheimer’s disease; PiD, classical Pick’s disease; FTLD, frontotemporal lobar dementia (Constantinidis type C); PSP, progressive supranuclear palsy; FTLD-GRNpos, frontotemporal lobar dementia with ubiquitin- and TDP-43-positive inclusions, progranulin mutation positive; FTLD-GRNneg, frontotemporal lobar dementia with ubiquitin- and TDP-43-positive inclusions, progranulin mutation negative.

Next, we carried out enrichment analysis of transcription factor (TF) targets using the ENCODE ChIP-Seq Significance Tool [56], which integrates data from hundreds of public ChIP-Seq data sets, to evaluate potential transcriptional regulators of the differentially expressed genes in the CNM. The 170 down-regulated CNM genes were significantly enriched (q-value < 0.05) for targets of six transcription factors: *REST, RBBP5, YY1, SIN3A, ZNF143* and *SP2*. The 73 up-regulated genes were significantly enriched (q-value < 0.05) for targets of three transcription factors: *IKZF1*, *STAT3* (in cells exposed to ethanol or tamoxifen), and *FOS* (in cells exposed to tamoxifen) (Figure 6A and 6B). As a significant portion of the CNM genes have expression levels correlated with normal aging, we repeated this analysis on the 545 and 449 genes positively and negatively correlated with aging, respectively. Unlike the CNM, no transcription factors were predicted for the genes negatively correlated with aging; however, the genes positively correlated with aging yielded an almost identical set of predicted transcription factors as the up-regulated component of the CNM, only including *POLR2A* instead of *IKZF1*. These findings suggest that genes up-regulated in both aging and neurodegeneration may share similar regulatory mechanisms, while those genes down-regulated in the CNM may be transcriptionally regulated in a manner unique to neurodegeneration.

Of the six transcription factors predicted to be upstream of the down-regulated CNM, *REST* and *YY1* have previously been implicated in neurodegeneration [88]-[92]. *REST* is a master regulator of neuronal genes, whose protein abundance increases with stress and aging, but decreases with AD, frontotemporal dementia and dementia with Lewy bodies [88]. *YY1* is a ubiquitous transcription factor previously noted to regulate several genes associated with neurodegenerative diseases including *BACE1* and *APP*[89], *SNCA*[90], *EAAT2*[91], *MTOR* and *PPARGC1A*[92].

### Assessment of disease-specific changes

**Figure 7:**
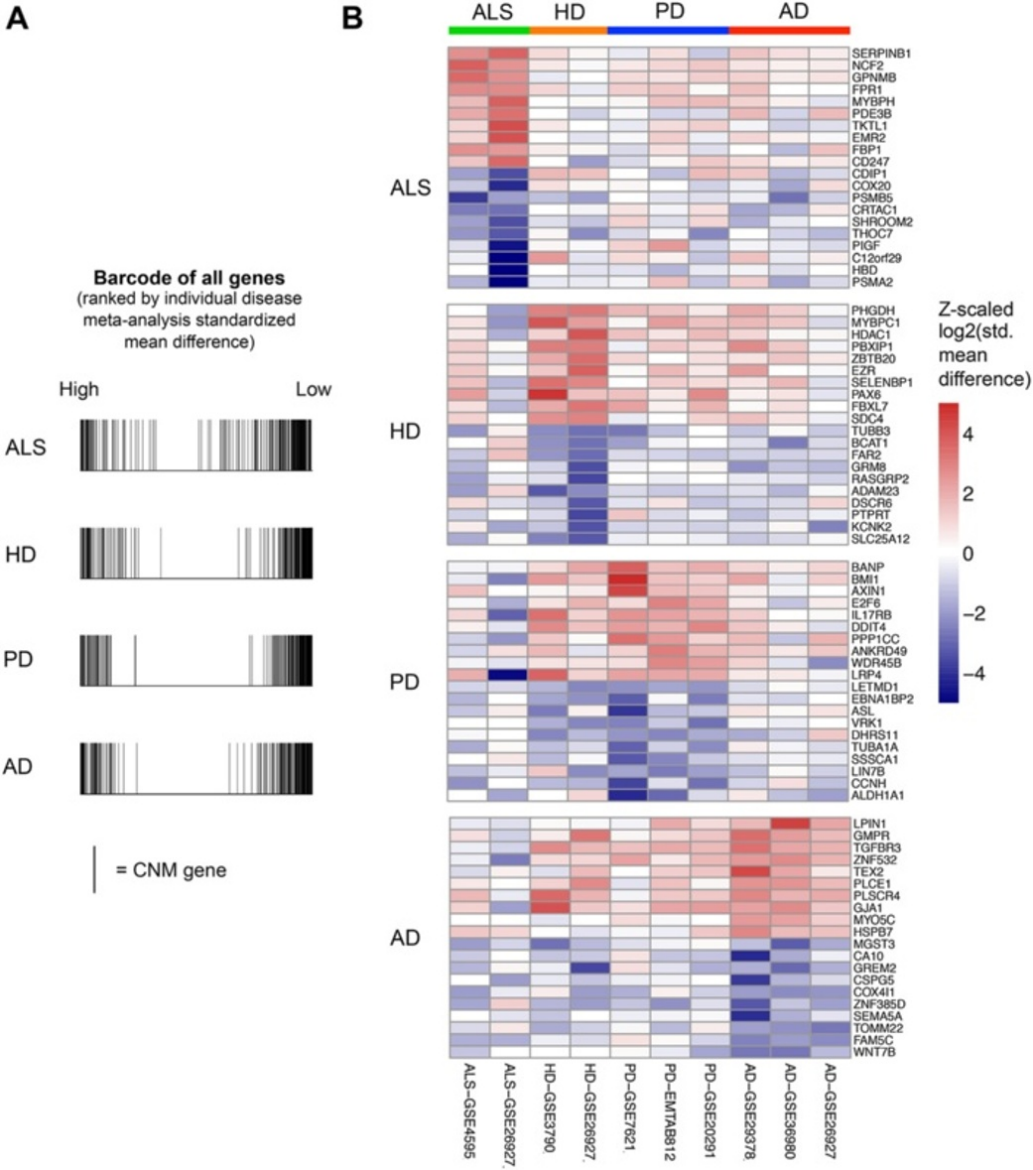
**Disease-specific meta-analysis. (A)** Distribution of the 243 CNM genes among individual disease meta-analysis gene lists. Each line represents the presence of a CNM gene among the 11,564 genes generated from disease-specific meta-analysis, ranked from most positive standardized mean difference (left) to most negative standardized mean difference (right). **(B)** Disease-specific meta-analysis, after removing genes differentially expressed across the other three diseases, identifies genes more strongly expressed in a single disease. Top 10 up-regulated and top 10 down-regulated genes shown. ALS, amyotrophic lateral sclerosis; HD, Huntington’s disease; PD, Parkinson’s disease; AD, Alzheimer’s disease.

As many elements of differential gene expression are shared across neurodegenerative diseases, we hypothesized that by removing elements common to other neurodegenerative diseases from a disease-specific gene signature and then functionally analyzing the remaining genes, we would be able to gain insights into the unique pathogenic mechanisms underlying each individual disease. Thus, for each disease, we used the meta-analysis approach to generate a rank ordered list of up- and down-regulated genes relative to controls. Examining where the CNM genes fall in this ordered list of genes for each individual disease validates that the CNM genes are similarly dysregulated in each neurodegenerative disease (Figure 7A). We then utilized the “leave-one-disease-out” meta-analyses previously generated (Figure 1), comprising the other 3 diseases (e.g. meta-analysis on HD alone vs. meta-analysis on AD, PD, and ALS together). These two analyses yielded ranked gene lists. We then removed significantly differentially expressed genes (FDR ≤ 0.05) identified from the 3-disease analysis gene list from the complete 1-disease analysis ranked gene list. We removed 1524, 780, 786 and 1019 genes from ALS, HD, PD, and AD-specific meta-analysis gene lists respectively. The shortened disease-specific gene lists represent genes that are expressed more strongly in a specific neurodegenerative disease (Figure 7B). As these genes represent disease-specific pathways, we then input these lists into GSEA PreRank for GO term enrichment analysis, as described earlier.

This analysis demonstrated significant unique up-regulation of immune and inflammatory genes in ALS specifically, including genes in the JAK-STAT cascade, suggesting additional inflammation over and above that shared by other neurodegenerative diseases. JAK-STAT genes have been found to be enriched in an independent gene expression analysis of ALS [93]. ALS and PD demonstrated down-regulation of additional proteasomal gene sets, and ALS showed down-regulation of genes involved in chomatin assembly suggestive of potentially unique epigenetic alterations. Notably, each of the 4 diseases, even after subtraction of genes significant in the other 3 disease, revealed persistently significant down-regulation of mitochondria-related genes, and all but ALS additionally revealed down-regulation of genes related to synaptic transmission—changes that were particularly prominent in AD (Additional file 1: Table S13).

## Discussion

**Figure 8:**
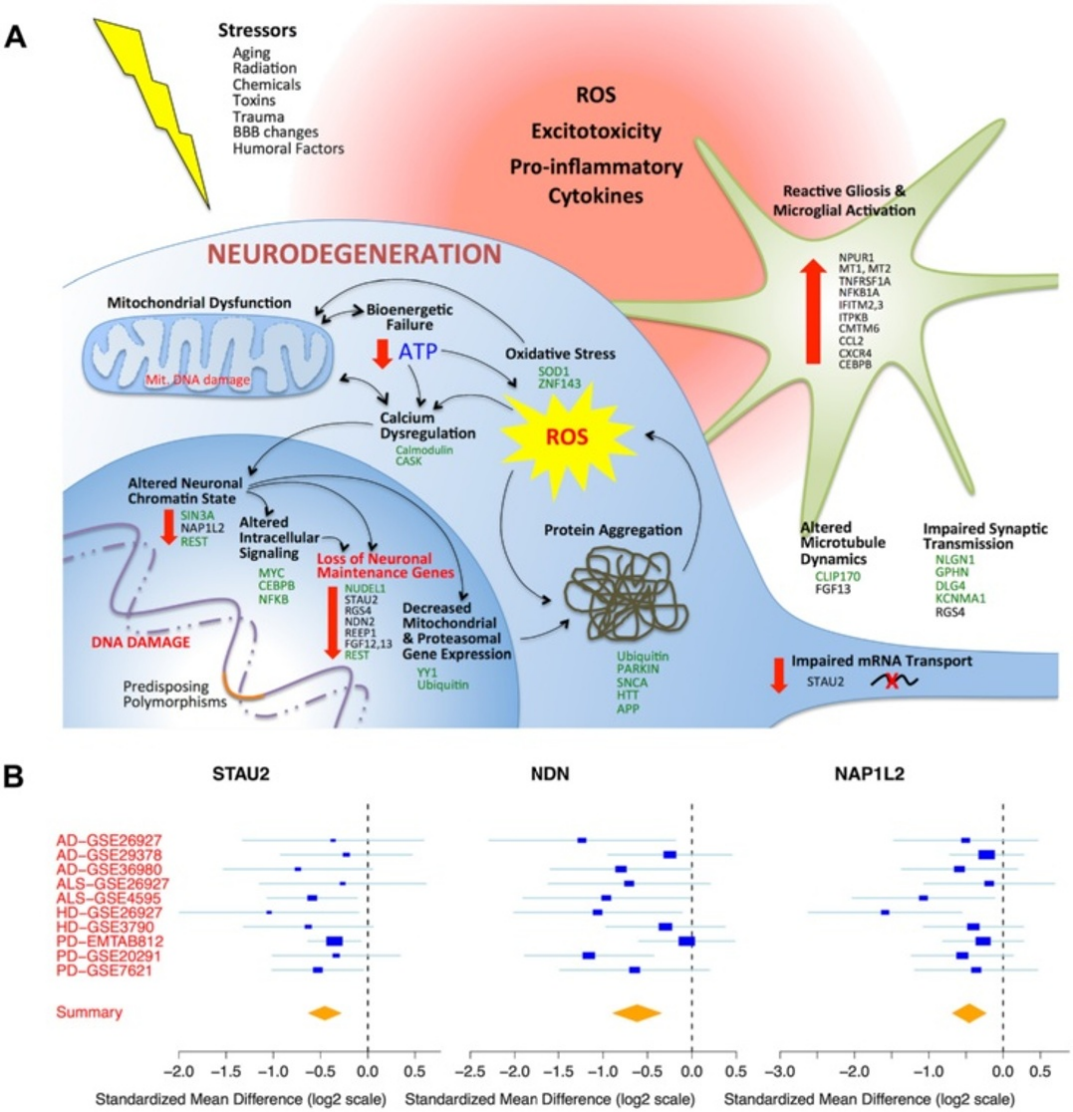
**Conserved elements of neurodegeneration. (A)** Schematic diagram of conserved elements of neurodegeneration. Select CNM genes of interest are shown in black text. Predicted hub genes and upstream transcription factors shown in green text. **(B)** Forest plots for highlighted candidate novel genes of interest. Forest plot x-axes show standardized mean difference (Hedges’ *g* in log2 scale) for genes in multiple data sets. Blue box sizes are inversely proportional to the SEM difference of the gene in each data set. Whiskers denote 95% confidence interval. Yellow diamonds represent combined mean difference for each gene. Yellow diamond width denotes 95% confidence interval.

Although each neurodegenerative disease has been studied in detail individually, no integrated analysis has previously determined what genes and pathways are consistently conserved across all neurodegenerative diseases (Figure 8A). In total, in this study we examined 31 separate patient cohorts consisting of 1,696 independent patient samples collected using various microarray platforms from diverse institutions in different countries to identify a robust and reproducible signature. Key findings from our analysis include: (1) a common signature of neurodegeneration that correlates with histologic disease severity and (2) identification of novel candidate convergent networks, hub proteins, and transcription factors for neurodegenerative diseases. We further analyzed expression of the CNM genes in normal aging brain to identify CNM genes that are altered in both aging and neurodegeneration, versus those altered in neurodegeneration alone. We also analyzed expression of the CNM genes enriched in specific cell types to better understand whether the changes in expression are due to changes in the number of specific functional transcripts or due to reduction in neuronal density. Our results identify down-regulation of genes important for neuronal maintenance and synaptic transmission, but relative preservation of most constitutively expressed neuronal genes. Finally, we performed disease-specific meta-analysis relative to common signatures of neurodegeneration. Although there are diverse genetic and environmental causes of different neurodegenerative disease processes, our results show that the CNM represents the most reproducibly convergent pathways. Furthermore, our unbiased approach validates that neurodegeneration commonly involves elements beyond neuroinflammation. As such, our data provide a valuable resource for interpreting disease mechanisms, connecting findings from one neurodegenerative disease to another, and driving novel hypotheses.

### Novel insights into mechanisms of neuronal degeneration

We identified 70 genes enriched in neurons that are decreased in the CNM, 53 of which are not altered with normal aging. Fewer than half of these 70 genes have previously been investigated for a specific role in neurodegeneration, despite substantial evidence in the literature suggesting that many of them could be of significant interest. We describe three examples, each of which is decreased in the CNM, but unchanged in aging, implying specificity to the neurodegenerative process (Figure 8B).

First, *STAU2*, a hub gene also identified in the MetaCore protein interaction analysis, is involved in neuronal RNA transport [80]. *STAU2* regulates the balance of neural stem cell maintenance versus differentiation during development [94], modulates long term depression by directing dendritic localization of protein synthesis in hippocampal neurons [95], and stabilizes the RNA of dendritic and synaptic proteins including *RGS4* (regulator of G protein signaling 4) [80]. Indeed *RGS4* itself was the second-most highly down-regulated gene in our entire meta-analysis (Figure 2) and has previously been associated with diseases ranging from AD [96] and HD [97], to schizophrenia [98] and depression [99]. Hence, given the role of *STAU2* in maintaining the fundamental structure of neuronal projections and synapses, pathologic decrease of *STAU2* expression could exacerbate neurodegeneration.

Second, necdin (*NDN*) is expressed predominantly in post-mitotic neurons where it forms a stable complex with p53 and sirtuin1 to down-regulate p53 acetylation and protect neurons from DNA damage-induced apoptosis [100]. Though an association with neurodegenerative disease has not previously been established, one study showed that necdin ablation in mice led to exacerbated dopaminergic cell loss after MPTP exposure, while overexpression of an AAV-necdin construct almost completely abrogated MPTP-induced dopaminergic cell loss [101]. These data suggest that *NDN* could be critical for maintaining neuronal resilience against exogenous stressors**.**

Third, *NAP1L2* promotes histone acetylation activity during neuronal differentiation [102]; *NAP1L2* mutants are embryonic lethal due to neural tube defects [103]. The importance of chromatin regulation in neurodegenerative disease was recently highlighted in experiments showing that Tau-induced heterochromatin loss results in aberrant gene expression in tauopathies [104]. However, it has previously been reported that brains with AD have a lower percentage of euchromatin than control brains [105]. Therefore, down-regulated *NAP1L2* would be consistent with the idea that neurodegeneration may additionally result from loss of essential regions of euchromatin, secondary to dysregulation of neuron-specific epigenetic regulators such as *NAP1L2*. If true, loss of *NAP1L2* could help to explain the diverse panel of down-regulated neuron-specific genes across neurodegenerative diseases.

These three genes are only a few from the list of neuron-associated CNM genes not previously associated with neurodegeneration. However, this list also contains numerous other potentially interesting candidates, including *FGF12*, a regulator of NFκB signaling in neurons [106]; *FGF13*, a microtubule stabilizing protein regulating neuronal polarization [107]; *MOAP1*, a modulator of apoptosis [108]; and *REEP1*, a gene involved in endoplasmic reticulum maintenance that is mutated in hereditary spastic paraplegia [109], among others. While several of these genes have established neurologic phenotypes in mutants, others are entirely unstudied.

### Common transcriptional regulators of neurodegenerative disease

We used the ENCODE ChIP-Seq significance tool to predict six transcription factors upstream of the 170 downregulated CNM genes. These transcription factors were not identified by our analysis of normal aging brain gene expression changes. Four of these have not previously been implicated in neurodegenerative diseases. *SIN3A* is a multifunctional scaffolding protein that forms part of a large co-repressor transcriptional regulatory complex. It recruits a wide variety of epigenetic modifiers that collectively repress gene expression in non-neuronal cells by regulating histone deacetylation and DNA methylation. In neurons, *SIN3A* works in concert with calcium-sensitive transcription factors to facilitate plasticity and activity-dependent gene regulation—processes fundamental to learning and memory [110]. *RBBP5* is a ubiquitously expressed transcriptional activator with histone methytransferase activity [111]. *RBBP5* co-purifies with the noncoding RNA 116HG, paternal deletion of which leads to Prader-Willi syndrome, a disease characterized in part by intellectual disability [112]. Furthermore, *RBBP5* has been implicated as a potential oncogene in glioblastoma [113]. *SP2* is a cell cycle regulator, deletion of which disrupts neurogenesis in embryonic and postnatal brain [114]. Interestingly, SP2 was the only predicted TF from the down-regulated CNM list whose mRNA level was decreased with aging. This suggests a potential dose-dependent effect of SP2 activity, with decreases in aging, and more severe decreases in neurodegeneration—both of which are associated with decreased neurogenesis. Finally, *ZNF143* plays roles in response to oxidative stress [115] and cell cycle regulation [116]. Collectively, these data identify potential transcriptional regulators of the core set of genes downregulated in neurodegeneration.

### Shared insights across neurodegenerative diseases

An important implication of our work is that prior insights gained regarding any of the genes in the CNM may prove broadly relevant across neurodegenerative diseases. For many genes in the CNM that have been associated with neurodegeneration previously, such a role has typically only been investigated for one or two diseases. However, such insights, when considering the convergent pathways revealed by our analysis, may benefit research in other diseases. For example, *RNF11* is a regulator of NF-κB signaling previously associated with only PD [117]. Low levels of axonal *NEFL* mRNA have previously been linked to ALS [118]. *RAB3B,* originally identified in a screen for genes enriched in MPTP-resistant A10 dopaminergic neurons relative to MPTP-susceptible A9 neurons, proved capable of protecting dopaminergic neurons when overexpressed in A9 neurons [119]. Neuronal *CD200* is a negative regulator of inflammation, previously found to be decreased AD [120] and PD [121]. Our data suggest that each of these genes is in fact decreased across each neurodegenerative diseases in our analysis—AD, PD, HD ALS, and likely others—suggesting broader potential applications of these prior findings.

### Metallothioneins and oxidative stress

Four of the top 10 up-regulated genes in the CNM were metallothioneins (*MT2A, MT1Z, MT1H*, and *MT1F*), each of which was also up-regulated in the normal aging brain. Metallothioneins are increasingly being explored in neurological diseases as potential therapeutic targets [122]. Metallothioneins 1 and 2 are expressed predominantly in astrocytes and are critical for buffering zinc, which has been implicated in the production of reactive oxygen species (ROS) in association with aging and inflammation [123]. States of increased oxidative stress promote mobilization of zinc from matrix metallothioneins after which they are taken up by mitochondria where they impede respiration and incite further ROS. This finding underscores the central additive role of further oxidative stress and mitochondrial dysfunction in all neurodegenerative diseases. That these genes were also up-regulated in normal aging in our analysis suggests that changes in these metallothioneins tended to be adaptive, rather than pathological in nature, which in turn suggests that astrocytes may attempt to mitigate this increased oxidative stress by further up-regulation of metallothioneins. Our identification of multiple reproducibly upregulated metallothioneins across all neurodegenerative diseases provides further impetus for further work in this area.

### Limitations

Despite its comprehensiveness, our analysis has several limitations. Although we have made inferences about genes likely altered in neurons, the resolution of cell-type specific data from mixed tissue is inherently limited. A number of methods for statistical deconvolution of mixed tissue gene expression data have been developed, which should be used to further explore cell-type specific expression in neurodegeneration once further human brain cell-specific gene expression profiles have been established [124]. Degeneration of specific neuronal subtypes in different diseases is believed to result from selective vulnerability—an issue that is not addressed in our analysis. Based on the use of microarray data, including that from multiple platforms, we can draw no conclusions regarding the broadly observed alterations in splice variants that are increasingly implicated in neurodegeneration; future analysis of transcriptome data derived from RNA-seq will illuminate this issue. Work to evaluate conserved epigenetic signatures of neurodegeneration will also be of great interest once sufficient relevant data are available in the future. Samples included in our analysis were largely derived from late stage disease, thereby masking potentially important early changes that could offer targets for preventative therapies. Nevertheless, the CNM was found to associate with histologic disease severity (Figure 3). Further work to collect region- and cell-type specific transcriptome data at multiple stages of disease with next generation sequencing technology will dramatically enhance the insights obtainable through bioinformatics analysis in the future.

In addition, while most included studies attempted to use brain tissue without co-pathologies, there are potentially other pathologies in the samples. Given the size of our study and the number of sources that the samples in these studies came from, we are optimistic that such confounding is minimized. Furthermore, the common neurodegeneration pathways identified may also be shared with other prevalent human diseases, like diabetes mellitus and atherosclerosis, which requires further investigation. However, such an analysis is out of the scope of our current analysis.

Finally, although our data shed light on the conserved signature of neurodegeneration, direct experimentation will be required to determine which of these newly highlighted changes are (1) direct etiological contributors to degeneration; (2) appropriate “survival” reactions activated in a valiant attempt to preserve cellular viability; or (3) stress-related changes that, though adaptive in the acute setting, lead to neurodegenerative sequelae in the long term.

## Conclusions

We carried out an integrated multi-cohort analysis of CNS tissue microarrays from AD, PD, HD, and ALS, thereby identifying a conserved transcriptional signature of neurodegeneration. These results were confirmed in additional independent publically available neurodegeneration CNS tissue microarray data sets meeting our inclusion criteria. Impaired bioenergetics with global down-regulation of mitochondria-related genes was the most predominantly conserved theme of neurodegeneration, accompanied by evidence of neuroinflammation, protein mishandling, oxidative stress, microglial activation, gliosis, and coordinated down-regulation of a host of genes essential for neurotransmission and normal neuronal function (Figure 8A). Overall, our functional analysis of the CNM, using Gene Ontology terms, MetaCore canonical pathways, and ChIP-Seq transcription factor prediction analysis, confirmed established findings and revealed additional novel insights. We believe the CNM represents a rich repository of convergent candidate genes that may be harnessed to improve our understanding of neurodegeneration, provide unique biomarkers for neurodegeneration, and facilitate the development of therapeutic strategies. We hope that these data will aid those studying neurodegeneration and pursuing therapies for these devastating diseases.

## Availability of supporting data

The data sets supporting the results of this article are available in the ArrayExpress and the Gene Expression Omnibus online repositories, at http://www.ebi.ac.uk/arrayexpress/ and http://www.ncbi.nlm.nih.gov/geo/. Data set and individual sample accession numbers can be found in Table 1 and Additional file 1: Table S1.

## Additional files

## Electronic supplementary material

Additional file 1: Table S1.: Gene expression data set sample accession IDs. **Table S2.** Gene expression meta-analysis gene list. **Table S3.** Common neurodegeneration module (CNM): pre-validated differentially expressed genes. **Table S4.** Common neurodegeneration module (CNM): validated differentially expressed genes. **Table S5.** Gene set enrichment analysis of the meta-analysis output for Gene Ontology (GO) terms. **Table S6.** MetaCore enrichment analysis. **Table S7.** MetaCore network analysis sub-network list. **Table S8.** Proteins detected to be over-connected with proteins corresponding to genes in the common neurodegeneration module (CNM). **Table S9.** Genes significantly correlated with aging in normal brain tissue. **Table S10.** Common neurodegeneration module (CNM): correlation with aging brain (upregulated component). **Table S11.** Common neurodegeneration module (CNM): correlation with aging brain (downregulated component). **Table S12.** Common neurodegeneration module (CNM): cell-type enriched genes. **Table S13.** Disease-specific gene set enrichment analysis after removal of genes significant in the other 3 diseases. (XLSX 905 KB)

Additional file 2: Figure S1.: CNM genes are consistently differentially expressed in samples in the discovery and validation data sets. **Figure S2.** CNM genes are consistently differentially expressed in samples in the secondary validation data sets. **Figure S3.** Complete annotations for Figure 4A. **Figure S4.** Forest plots for highlighted hub genes of interest. **Figure S5.** Network and pathway analyses reveal common pathways and hubs in neurodegeneration. **Figure S6.** Heat map of CNM genes and their correlation with aging in three independent aging brain data sets. (PDF 13 MB)

Below are the links to the authors’ original submitted files for images.

Authors’ original file for figure 1

Authors’ original file for figure 2

Authors’ original file for figure 3

Authors’ original file for figure 4

Authors’ original file for figure 5

Authors’ original file for figure 6

Authors’ original file for figure 7

Authors’ original file for figure 8

## Abbreviations

AD: Alzheimer’s disease
ALS: Amyotrophic lateral sclerosis
CNM: Common neurodegeneration module
CNS: Central nervous system
DAVID: Database for annotation, visualization and Integrated discovery
FDR: False discovery rate
FTLD: Frontotemporal lobar dementia
GEXC: Gene expression commons
GO: Gene ontology
GSEA: Gene set enrichment analysis
HD: Huntington’s disease
MSigDB: Molecular signatures database
PD: Parkinson’s disease
TF: Transcription factor

## References

[1] Dorsey ER, George BP, Leff B, Willis AW (2013). The coming crisis: obtaining care for the growing burden of neurodegenerative conditions. Neurology.

[2] Zlokovic BV (2011). Neurovascular pathways to neurodegeneration in Alzheimer’s disease and other disorders. Nat Rev Neurosci.

[3] Mehta A, Prabhakar M, Kumar P, Deshmukh R, Sharma PL (2013). Excitotoxicity: bridge to various triggers in neurodegenerative disorders. Eur J Pharmacol.

[4] Uttara B, Singh AV, Zamboni P, Mahajan RT (2009). Oxidative stress and neurodegenerative diseases: a review of upstream and downstream antioxidant therapeutic options. Curr Neuropharmacol.

[5] Masel BE, DeWitt DS (2010). Traumatic brain injury: a disease process, not an event. J Neurotrauma.

[6] Migliore L, Coppedé F (2009). Environmental-induced oxidative stress in neurodegenerative disorders and aging. Mutat Res.

[7] Chen H, Chan DC (2009). Mitochondrial dynamics–fusion, fission, movement, and mitophagy–in neurodegenerative diseases. Hum Mol Genet.

[8] Lagier-Tourenne C, Polymenidou M, Cleveland DW (2010). TDP-43 and FUS/TLS: emerging roles in RNA processing and neurodegeneration. Hum Mol Genet.

[9] Wong E, Cuervo AM (2010). Autophagy gone awry in neurodegenerative diseases. Nat Neurosci.

[10] Moreno JA, Halliday M, Molloy C, Radford H, Verity N, Axten JM, Ortori CA, Willis AE, Fischer PM, Barrett DA, Mallucci GR (2013). Oral treatment targeting the unfolded protein response prevents neurodegeneration and clinical disease in prion-infected mice. Sci Transl Med.

[11] Cooper-Knock J, Kirby J, Ferraiuolo L, Heath PR, Rattray M, Shaw PJ (2012). Gene expression profiling in human neurodegenerative disease. Nat Rev Neurol.

[12] Ramanan VK, Saykin AJ (2013). Pathways to neurodegeneration: mechanistic insights from GWAS in Alzheimer’s disease, Parkinson's disease, and related disorders. Am J Neurodegener Dis.

[13] Vogt IR, Lees AJ, Evert BO, Klockgether T, Bonin M, Wüllner U (2006). Transcriptional changes in multiple system atrophy and Parkinson’s disease putamen. Exp Neurol.

[14] Bossers K, Wirz KTS, Meerhoff GF, Essing AHW, van Dongen JW, Houba P, Kruse CG, Verhaagen J, Swaab DF (2010). Concerted changes in transcripts in the prefrontal cortex precede neuropathology in Alzheimer’s disease. Brain.

[15] Blalock EM, Buechel HM, Popovic J, Geddes JW, Landfield PW (2011). Microarray analyses of laser-captured hippocampus reveal distinct gray and white matter signatures associated with incipient Alzheimer’s disease. J Chem Neuroanat.

[16] Zheng B, Liao Z, Locascio JJ, Lesniak KA, Roderick SS, Watt ML, Eklund AC, Zhang-James Y, Kim PD, Hauser MA, Grünblatt E, Moran LB, Mandel SA, Riederer P, Miller RM, Federoff HJ, Wüllner U, Papapetropoulos S, Youdim MB, Cantuti-Castelvetri I, Young AB, Vance JM, Davis RL, Hedreen JC, Adler CH, Beach TG, Graeber MB, Middleton FA, Rochet J-C, Scherzer CR (2010). PGC-1α, a potential therapeutic target for early intervention in Parkinson’s disease. Sci Transl Med.

[17] Rhodes DR, Yu J, Shanker K, Deshpande N, Varambally R, Ghosh D, Barrette T, Pandey A, Chinnaiyan AM (2004). Large-scale meta-analysis of cancer microarray data identifies common transcriptional profiles of neoplastic transformation and progression. Proc Natl Acad Sci U S A.

[18] Jenner RG, Young R (2005). Insights into host responses against pathogens from transcriptional profiling. Nat Rev Microbiol.

[19] Chen R, Khatri P, Mazur PK, Polin M, Zheng Y, Vaka D, Hoang CD, Shrager J, Xu Y, Vicent S, Butte A, Sweet-Cordero EA: A meta-analysis of lung cancer gene expression identifies PTK7 as a survival gene in lung adenocarcinoma. *Cancer Res* 2014, doi:10.1158/0008–5472.CAN-13–2775. 10.1158/0008-5472.CAN-13-2775 PMC4084668 24654231

[20] Khatri P, Roedder S, Kimura N, De Vusser K, Morgan A, Gong Y, Fischbein MP, Robbins RC, Naesens M, Butte AJ, Sarwal MM (2013). A common rejection module (CRM) for acute rejection across multiple organs identifies novel therapeutics for organ transplantation. J Exp Med.

[21] Kolde R: pheatmap: Pretty Heatmaps.., [http://cran.r-project.org/web/packages/pheatmap/index.html]

[22] Lederer CW, Torrisi A, Pantelidou M, Santama N, Cavallaro S (2007). Pathways and genes differentially expressed in the motor cortex of patients with sporadic amyotrophic lateral sclerosis. BMC Genomics.

[23] Durrenberger PF, Fernando FS, Magliozzi R, Kashefi SN, Bonnert TP, Ferrer I, Seilhean D, Nait-Oumesmar B, Schmitt A, Gebicke-Haerter PJ, Falkai P, Grünblatt E, Palkovits M, Parchi P, Capellari S, Arzberger T, Kretzschmar H, Roncaroli F, Dexter DT, Reynolds R (2012). Selection of novel reference genes for use in the human central nervous system: a BrainNet Europe Study. Acta Neuropathol.

[24] Hodges A, Strand AD, Aragaki AK, Kuhn A, Sengstag T, Hughes G, Elliston LA, Hartog C, Goldstein DR, Thu D, Hollingsworth ZR, Collin F, Synek B, Holmans PA, Young AB, Wexler NS, Delorenzi M, Kooperberg C, Augood SJ, Faull RLM, Olson JM, Jones L, Luthi-Carter R (2006). Regional and cellular gene expression changes in human Huntington’s disease brain. Hum Mol Genet.

[25] Lesnick TG, Papapetropoulos S, Mash DC, Ffrench-Mullen J, Shehadeh L, de Andrade M, Henley JR, Rocca WA, Ahlskog JE, Maraganore DM (2007). A genomic pathway approach to a complex disease: axon guidance and Parkinson disease. PLoS Genet.

[26] Dumitriu A, Latourelle JC, Hadzi TC, Pankratz N, Garza D, Miller JP, Vance JM, Foroud T, Beach TG, Myers RH (2012). Gene expression profiles in Parkinson disease prefrontal cortex implicate FOXO1 and genes under its transcriptional regulation. PLoS Genet.

[27] Zhang Y, James M, Middleton FA, Davis RL (2005). Transcriptional analysis of multiple brain regions in Parkinson’s disease supports the involvement of specific protein processing, energy metabolism, and signaling pathways, and suggests novel disease mechanisms. Am J Med Genet B Neuropsychiatr Genet.

[28] Miller JA, Woltjer RL, Goodenbour JM, Horvath S, Geschwind DH (2013). Genes and pathways underlying regional and cell type changes in Alzheimer’s disease. Genome Med.

[29] Hokama M, Oka S, Leon J, Ninomiya T, Honda H, Sasaki K, Iwaki T, Ohara T, Sasaki T, Laferla FM, Kiyohara Y, Nakabeppu Y (2013). Altered Expression of Diabetes-Related Genes in Alzheimer’s Disease Brains: The Hisayama Study.

[30] Webster JA, Gibbs JR, Clarke J, Ray M, Zhang W, Holmans P, Rohrer K, Zhao A, Marlowe L, Kaleem M, McCorquodale DS, Cuello C, Leung D, Bryden L, Nath P, Zismann VL, Joshipura K, Huentelman MJ, Hu-Lince D, Coon KD, Craig DW, Pearson JV, Heward CB, Reiman EM, Stephan D, Hardy J, Myers AJ (2009). Genetic control of human brain transcript expression in Alzheimer disease. Am J Hum Genet.

[31] Grünblatt E, Mandel S, Jacob-Hirsch J, Zeligson S, Amariglo N, Rechavi G, Li J, Ravid R, Roggendorf W, Riederer P, Youdim MBH (2004). Gene expression profiling of parkinsonian substantia nigra pars compacta; alterations in ubiquitin-proteasome, heat shock protein, iron and oxidative stress regulated proteins, cell adhesion/cellular matrix and vesicle trafficking genes. J Neural Transm.

[32] Hauser MA, Li Y-J, Xu H, Noureddine MA, Shao YS, Gullans SR, Scherzer CR, Jensen RV, McLaurin AC, Gibson JR, Scott BL, Jewett RM, Stenger JE, Schmechel DE, Hulette CM, Vance JM (2005). Expression profiling of substantia nigra in Parkinson disease, progressive supranuclear palsy, and frontotemporal dementia with parkinsonism. Arch Neurol.

[33] Moran LB, Duke DC, Deprez M, Dexter DT, Pearce RKB, Graeber MB (2006). Whole genome expression profiling of the medial and lateral substantia nigra in Parkinson’s disease. Neurogenetics.

[34] Lewandowski NM, Ju S, Verbitsky M, Ross B, Geddie ML, Rockenstein E, Adame A, Muhammad A, Vonsattel JP, Ringe D, Cote L, Lindquist S, Masliah E, Petsko GA, Marder K, Clark LN, Small SA (2010). Polyamine pathway contributes to the pathogenesis of Parkinson disease. Proc Natl Acad Sci U S A.

[35] Blalock EM, Geddes JW, Chen KC, Porter NM, Markesbery WR, Landfield PW (2004). Incipient Alzheimer’s disease: microarray correlation analyses reveal major transcriptional and tumor suppressor responses. Proc Natl Acad Sci U S A.

[36] Bronner IF, Bochdanovits Z, Rizzu P, Kamphorst W, Ravid R, van Swieten JC, Heutink P (2009). Comprehensive mRNA expression profiling distinguishes tauopathies and identifies shared molecular pathways. PLoS One.

[37] Chen-Plotkin AS, Geser F, Plotkin JB, Clark CM, Kwong LK, Yuan W, Grossman M, Van Deerlin VM, Trojanowski JQ, Lee VM-Y (2008). Variations in the progranulin gene affect global gene expression in frontotemporal lobar degeneration. Hum Mol Genet.

[38] Benjamini Y, Hochberg Y (1995). Controlling the false discovery rate: a practical and powerful approach to multiple testing. J R Stat Soc Ser B.

[39] Zhang B, Gaiteri C, Bodea L-G, Wang Z, McElwee J, Podtelezhnikov A, Zhang C, Xie T, Tran L, Dobrin R, Fluder E, Clurman B, Melquist S, Narayanan M, Suver C, Shah H, Mahajan M, Gillis T, Mysore J, MacDonald ME, Lamb JR, Bennett D, Molony C, Stone DJ, Gudnason V, Myers AJ, Schadt EE, Neumann H, Zhu J, Emilsson V (2013). Integrated systems approach identifies genetic nodes and networks in late-onset Alzheimer’s disease. Cell.

[40] Wickham H (2009). ggplot2: Elegant Graphics for Data Analysis.

[41] Subramanian A, Tamayo P, Mootha VK, Mukherjee S, Ebert BL, Gillette MA, Paulovich A, Pomeroy SL, Golub TR, Lander ES, Mesirov JP (2005). Gene set enrichment analysis: a knowledge-based approach for interpreting genome-wide expression profiles. Proc Natl Acad Sci U S A.

[42] Merico D, Isserlin R, Bader GD (2011). Network Biology.

[43] Saito R, Smoot ME, Ono K, Ruscheinski J, Wang P, Lotia S, Pico AR, Bader GD, Ideker T (2012). A travel guide to Cytoscape plugins. Nat Methods.

[44] Berchtold NC, Cribbs DH, Coleman PD, Rogers J, Head E, Kim R, Beach T, Miller C, Troncoso J, Trojanowski JQ, Zielke HR, Cotman CW (2008). Gene expression changes in the course of normal brain aging are sexually dimorphic. Proc Natl Acad Sci U S A.

[45] Lu T, Pan Y, Kao S-Y, Li C, Kohane I, Chan J, Yankner BA (2004). Gene regulation and DNA damage in the ageing human brain. Nature.

[46] Colantuoni C, Lipska BK, Ye T, Hyde TM, Tao R, Leek JT, Colantuoni EA, Elkahloun AG, Herman MM, Weinberger DR, Kleinman JE (2011). Temporal dynamics and genetic control of transcription in the human prefrontal cortex. Nature.

[47] McLeod AI:Kendall R Package.., [http://cran.r-project.org/web/packages/Kendall/index.html]

[48] Huang DW, Sherman BT, Lempicki R (2009). Systematic and integrative analysis of large gene lists using DAVID bioinformatics resources. Nat Protoc.

[49] Cahoy JD, Emery B, Kaushal A, Foo LC, Zamanian JL, Christopherson KS, Xing Y, Lubischer JL, Krieg P, Krupenko S, Thompson WJ, Barres B (2008). A transcriptome database for astrocytes, neurons, and oligodendrocytes: a new resource for understanding brain development and function. J Neurosci.

[50] Doyle JP, Dougherty JD, Heiman M, Schmidt EF, Stevens TR, Ma G, Bupp S, Shrestha P, Shah RD, Doughty ML, Gong S, Greengard P, Heintz N (2008). Application of a translational profiling approach for the comparative analysis of CNS cell types. Cell.

[51] Anandasabapathy N, Victora GD, Meredith M, Feder R, Dong B, Kluger C, Yao K, Dustin ML, Nussenzweig MC, Steinman RM, Liu K (2011). Flt3L controls the development of radiosensitive dendritic cells in the meninges and choroid plexus of the steady-state mouse brain. J Exp Med.

[52] Seita J, Sahoo D, Rossi DJ, Bhattacharya D, Serwold T, Inlay M, Ehrlich LIR, Fathman JW, Dill DL, Weissman IL (2012). Gene Expression Commons: an open platform for absolute gene expression profiling. PLoS One.

[53] Zamanian JL, Xu L, Foo LC, Nouri N, Zhou L, Giffard RG, Barres B (2012). Genomic analysis of reactive astrogliosis. J Neurosci.

[54] Xue J, Schmidt SV, Sander J, Draffehn A, Krebs W, Quester I, De Nardo D, Gohel TD, Emde M, Schmidleithner L, Ganesan H, Nino-castro A, Mallmann MR, Labzin L, Theis H, Kraut M, Beyer M, Latz E, Freeman TC, Ulas T, Schultze JL (2014). Resource Transcriptome-Based Network Analysis Reveals a Spectrum Model of Human Macrophage Activation. Immunity.

[55] Chiu IM, Morimoto ET, Goodarzi H, Liao JT, O’Keeffe S, Phatnani HP, Muratet M, Carroll MC, Levy S, Tavazoie S, Myers RM, Maniatis T (2013). A neurodegeneration-specific gene-expression signature of acutely isolated microglia from an amyotrophic lateral sclerosis mouse model. Cell Rep.

[56] Auerbach RK, Chen B, Butte AJ (2013). Relating genes to function: identifying enriched transcription factors using the ENCODE ChIP-Seq significance tool. Bioinformatics.

[57] Strohmeyer R, Shelton J, Lougheed C, Breitkopf T (2014). CCAAT-enhancer binding protein-β expression and elevation in Alzheimer’s disease and microglial cell cultures. PLoS One.

[58] Valente T, Mancera P, Tusell JM, Serratosa J, Saura J (2012). C/EBPβ expression in activated microglia in amyotrophic lateral sclerosis. Neurobiol Aging.

[59] Cheung ZH, Ip NY (2012). Cdk5: a multifaceted kinase in neurodegenerative diseases. Trends Cell Biol.

[60] Marambaud P, Dreses-Werringloer U, Vingtdeux V (2009). Calcium signaling in neurodegeneration. Mol Neurodegener.

[61] Huang C, Hatzi K, Melnick A (2013). Lineage-specific functions of Bcl-6 in immunity and inflammation are mediated by distinct biochemical mechanisms. Nat Immunol.

[62] Hamilton JA (2008). Colony-stimulating factors in inflammation and autoimmunity. Nat Rev Immunol.

[63] Izikson L, Klein RS, Charo IF, Weiner HL, Luster AD (2000). Resistance to experimental autoimmune encephalomyelitis in mice lacking the CC chemokine receptor (CCR)2. J Exp Med.

[64] Fehrenbacher JC, Taylor CP, Vasko MR (2003). Pregabalin and gabapentin reduce release of substance P and CGRP from rat spinal tissues only after inflammation or activation of protein kinase C. Pain.

[65] Citron B, Dennis JS, Zeitlin RS, Echeverria V (2008). Transcription factor Sp1 dysregulation in Alzheimer’s disease. J Neurosci Res.

[66] Ryu H, Lee J, Zaman K, Kubilis J, Ferrante RJ, Ross BD, Neve R, Ratan RR (2003). Sp1 and Sp3 are oxidative stress-inducible, antideath transcription factors in cortical neurons. J Neurosci.

[67] Heicklen-Klein A, Ginzburg I (2000). Tau promoter confers neuronal specificity and binds Sp1 and AP-2. J Neurochem.

[68] Dantzer R, O’Connor JC, Freund GG, Johnson RW, Kelley KW (2008). From inflammation to sickness and depression: when the immune system subjugates the brain. Nat Rev Neurosci.

[69] Chen Y, Fu AKY, Ip NY (2012). Eph receptors at synapses: implications in neurodegenerative diseases. Cell Signal.

[70] Lin MT, Beal MF (2006). Mitochondrial dysfunction and oxidative stress in neurodegenerative diseases. Nature.

[71] Sindi I, Tannenberg RK, Dodd PR (2014). A role for the neurexin-neuroligin complex in Alzheimer’s disease. Neurobiol Aging.

[72] Lee M (2013). Neurotransmitters and microglial-mediated neuroinflammation. Curr Protein Pept Sci.

[73] Tanaka K, Matsuda N (2014). Proteostasis and neurodegeneration: the roles of proteasomal degradation and autophagy. Biochim Biophys Acta.

[74] McShea A, Lee H, Petersen RB, Casadesus G, Vincent I, Linford NJ, Funk J-O, Shapiro RA, Smith MA (2007). Neuronal cell cycle re-entry mediates Alzheimer disease-type changes. Biochim Biophys Acta.

[75] Willis M, Leitner I, Jellinger KA, Marksteiner J (2011). Chromogranin peptides in brain diseases. J Neural Transm.

[76] Kashiwaya K, Hosokawa M, Eguchi H, Ohigashi H, Ishikawa O, Shinomura Y, Nakamura Y, Nakagawa H (2009). Identification of C2orf18, termed ANT2BP (ANT2-binding protein), as one of the key molecules involved in pancreatic carcinogenesis. Cancer Sci.

[77] Caccamo D, Condello S, Ferlazzo N, Curròò M, Griffin M, Ientile R (2013). Transglutaminase 2 interaction with small heat shock proteins mediate cell survival upon excitotoxic stress. Amino Acids.

[78] Shigenari A, Ando A, Baba T, Yamamoto T, Katsuoka Y, Inoko H (1998). Characterization of alkaline phosphatase genes expressed in seminoma by cDNA cloning. Cancer Res.

[79] Bosson A, Soleilhac J-M, Valiron O, Job D, Andrieux A, Moutin M-J (2012). Cap-Gly proteins at microtubule plus ends: is EB1 detyrosination involved?. PLoS One.

[80] Heraud-Farlow JE, Sharangdhar T, Li X, Pfeifer P, Tauber S, Orozco D, Hörmann A, Thomas S, Bakosova A, Farlow AR, Edbauer D, Lipshitz HD, Morris QD, Bilban M, Doyle M, Kiebler M (2013). Staufen2 regulates neuronal target RNAs. Cell Rep.

[81] Chansard M, Hong J-H, Park Y-U, Park SK, Nguyen MD (2011). Ndel1, Nudel (Noodle): flexible in the cell?. Cytoskeleton (Hoboken).

[82] Scott C, Keating L, Bellamy M, Baines AJ (2001). Protein 4.1 in forebrain postsynaptic density preparations: enrichment of 4.1 gene products and detection of 4.1R binding proteins. Eur J Biochem.

[83] Hong H, Yang L, Stallcup MR (1999). Hormone-independent transcriptional activation and coactivator binding by novel orphan nuclear receptor ERR3. J Biol Chem.

[84] Hu H, Shao LR, Chavoshy S, Gu N, Trieb M, Behrens R, Laake P, Pongs O, Knaus HG, Ottersen OP, Storm JF (2001). Presynaptic Ca2+−activated K+ channels in glutamatergic hippocampal terminals and their role in spike repolarization and regulation of transmitter release. J Neurosci.

[85] Sloane JA, Hollander W, Moss MB, Rosene DL, Abraham CR (1999). Increased microglial activation and protein nitration in white matter of the aging monkey. Neurobiol Aging.

[86] Norris CM, Halpain S, Foster TC (1998). Reversal of age-related alterations in synaptic plasticity by blockade of L-type Ca2+ channels. J Neurosci.

[87] Persson J, Nyberg L, Lind J, Larsson A, Nilsson L-G, Ingvar M, Buckner RL (2006). Structure-function correlates of cognitive decline in aging. Cereb Cortex.

[88] Lu T, Aron L, Zullo J, Pan Y, Kim H, Chen Y, Yang T-H, Kim H-M, Drake D, Liu XS, Bennett DA, Colaiácovo MP, Yankner BA (2014). REST and stress resistance in ageing and Alzheimer’s disease. Nature.

[89] Lahiri DK, Ge Y-W, Rogers JT, Sambamurti K, Greig NH, Maloney B (2006). Taking down the unindicted co-conspirators of amyloid beta-peptide-mediated neuronal death: shared gene regulation of BACE1 and APP genes interacting with CREB, Fe65 and YY1 transcription factors. Curr Alzheimer Res.

[90] Mizuta I, Takafuji K, Ando Y, Satake W, Kanagawa M, Kobayashi K, Nagamori S, Shinohara T, Ito C, Yamamoto M, Hattori N, Murata M, Kanai Y, Murayama S, Nakagawa M, Toda T (2013). YY1 binds to α-synuclein 3’-flanking region SNP and stimulates antisense noncoding RNA expression. J Hum Genet.

[91] Karki P, Webb A, Smith K, Johnson J, Lee K, Son D-S, Aschner M, Lee E (2014). Yin Yang 1 Is a Repressor of Glutamate Transporter EAAT2, and It Mediates Manganese-Induced Decrease of EAAT2 Expression in Astrocytes. Mol Cell Biol.

[92] Cunningham JT, Rodgers JT, Arlow DH, Vazquez F, Mootha VK, Puigserver P (2007). mTOR controls mitochondrial oxidative function through a YY1-PGC-1alpha transcriptional complex. Nature.

[93] Figueroa-Romero C, Hur J, Bender DE, Delaney CE, Cataldo MD, Smith AL, Yung R, Ruden DM, Callaghan BC, Feldman EL (2012). Identification of epigenetically altered genes in sporadic amyotrophic lateral sclerosis. PLoS One.

[94] Vessey JP, Amadei G, Burns SE, Kiebler MA, Kaplan DR, Miller FD (2012). An asymmetrically localized Staufen2-dependent RNA complex regulates maintenance of mammalian neural stem cells. Cell Stem Cell.

[95] Lebeau G, Miller LC, Tartas M, McAdam R, Laplante I, Badeaux F, DesGroseillers L, Sossin WS, Lacaille J-C (2011). Staufen 2 regulates mGluR long-term depression and Map1b mRNA distribution in hippocampal neurons. Learn Mem.

[96] Emilsson L, Saetre P, Jazin E (2006). Low mRNA levels of RGS4 splice variants in Alzheimer’s disease: association between a rare haplotype and decreased mRNA expression. Synapse.

[97] Runne H, Régulier E, Kuhn A, Zala D, Gokce O, Perrin V, Sick B, Aebischer P, Déglon N, Luthi-Carter R (2008). Dysregulation of gene expression in primary neuron models of Huntington’s disease shows that polyglutamine-related effects on the striatal transcriptome may not be dependent on brain circuitry. J Neurosci.

[98] Mirnics K, Middleton FA, Stanwood GD, Lewis DA, Levitt P (2001). Disease-specific changes in regulator of G-protein signaling 4 (RGS4) expression in schizophrenia. Mol Psychiatry.

[99] Guilloux J-P, Douillard-Guilloux G, Kota R, Wang X, Gardier AM, Martinowich K, Tseng GC, Lewis DA, Sibille E (2012). Molecular evidence for BDNF- and GABA-related dysfunctions in the amygdala of female subjects with major depression. Mol Psychiatry.

[100] Hasegawa K, Yoshikawa K (2008). Necdin regulates p53 acetylation via Sirtuin1 to modulate DNA damage response in cortical neurons. J Neurosci.

[101] Yasuda T, Yoshikawa K, Przedborski S, Mizuno Y, Mochizuki H (2012). Cell cycle regulation promotes survival of dopaminergic neurons in experimental Parkinson’s disease [abstract]. Mov Disord.

[102] Attia M, Rachez C, De Pauw A, Avner P, Rogner UC (2007). Nap1l2 promotes histone acetylation activity during neuronal differentiation. Mol Cell Biol.

[103] Rogner UC, Spyropoulos DD, Le Novére N, Changeux JP, Avner P (2000). Control of neurulation by the nucleosome assembly protein-1-like 2. Nat Genet.

[104] Frost B, Hemberg M, Lewis J, Feany MB (2014). Tau promotes neurodegeneration through global chromatin relaxation. Nat Neurosci.

[105] Crapper DR, Quittkat S, de Boni U (1979). Altered chromatin conformation in Alzheimer’s disease. Brain.

[106] König H-G, Fenner BJ, Byrne JC, Schwamborn RF, Bernas T, Jefferies CA, Prehn JHM (2012). Fibroblast growth factor homologous factor 1 interacts with NEMO to regulate NF-κB signaling in neurons. J Cell Sci.

[107] Wu Q-F, Yang L, Li S, Wang Q, Yuan X-B, Gao X, Bao L, Zhang X (2012). Fibroblast growth factor 13 is a microtubule-stabilizing protein regulating neuronal polarization and migration. Cell.

[108] Fu NY, Sukumaran SK, Yu VC (2007). Inhibition of ubiquitin-mediated degradation of MOAP-1 by apoptotic stimuli promotes Bax function in mitochondria. Proc Natl Acad Sci U S A.

[109] Züchner S, Wang G, Tran-Viet K-N, Nance MA, Gaskell PC, Vance JM, Ashley-Koch AE, Pericak-Vance MA (2006). Mutations in the novel mitochondrial protein REEP1 cause hereditary spastic paraplegia type 31. Am J Hum Genet.

[110] Schoch H, Abel T (2014). Transcriptional co-repressors and memory storage. Neuropharmacology.

[111] Dou Y, Milne TA, Ruthenburg AJ, Lee S, Lee JW, Verdine GL, Allis CD, Roeder RG (2006). Regulation of MLL1 H3K4 methyltransferase activity by its core components. Nat Struct Mol Biol.

[112] Powell WT, Coulson RL, Crary FK, Wong SS, Ach RA, Tsang P, Alice Yamada N, Yasui DH, Lasalle JM (2013). A Prader-Willi locus lncRNA cloud modulates diurnal genes and energy expenditure. Hum Mol Genet.

[113] Bralten LBC, Kloosterhof NK, Gravendeel LAM, Sacchetti A, Duijm EJ, Kros JM, van den Bent MJ, Hoogenraad CC, Sillevis Smitt PAE, French PJ (2010). Integrated genomic profiling identifies candidate genes implicated in glioma-genesis and a novel LEO1-SLC12A1 fusion gene. Genes Chromosomes Cancer.

[114] Liang H, Xiao G, Yin H, Hippenmeyer S, Horowitz JM, Ghashghaei HT (2013). Neural development is dependent on the function of specificity protein 2 in cell cycle progression. Development.

[115] Dhawan D, Sharma RR, Sharma R, Dash RJ (1988). Effect of short-term and long-term lithium treatment on uptake and retention of iodine-131 in rat thyroid. Aust J Biol Sci.

[116] Izumi H, Yasuniwa Y, Akiyama M, Yamaguchi T, Kuma A, Kitamura N, Kohno K (2011). Forced Expression of ZNF143 Restrains Cancer Cell Growth. Cancers (Basel).

[117] Pranski E, Van Sanford CD, Dalal N, Orr AL, Karmali D, Cooper DS, Gearing M, Lah JJ, Levey AI, Betarbet R (2013). NF-κB activity is inversely correlated to RNF11 expression in Parkinson’s disease. Neurosci Lett.

[118] Ishtiaq M, Campos-Melo D, Volkening K, Strong MJ (2014). Analysis of novel NEFL mRNA targeting microRNAs in amyotrophic lateral sclerosis. PLoS One.

[119] Chung CY, Koprich JB, Hallett PJ, Isacson O (2009). Functional enhancement and protection of dopaminergic terminals by RAB3B overexpression. Proc Natl Acad Sci U S A.

[120] Walker DG, Dalsing-Hernandez JE, Campbell NA, Lue L-F (2009). Decreased expression of CD200 and CD200 receptor in Alzheimer’s disease: a potential mechanism leading to chronic inflammation. Exp Neurol.

[121] Wang X-J, Ye M, Zhang Y-H, Chen S-D (2007). CD200-CD200R regulation of microglia activation in the pathogenesis of Parkinson’s disease. J Neuroimmune Pharmacol.

[122] Bolognin S, Cozzi B, Zambenedetti P, Zatta P (2014). Metallothioneins and the Central Nervous System: From a Deregulation in Neurodegenerative Diseases to the Development of New Therapeutic Approaches. J Alzheimers Dis.

[123] Frazzini V, Rockabrand E, Mocchegiani E, Sensi SL: Oxidative stress and brain aging: is zinc the link? *Biogerontology* 7: 307–314. doi:10.1007/s10522–006–9045–7. 10.1007/s10522-006-9045-7 17028932

[124] Shen-Orr SS, Gaujoux R (2013). Computational deconvolution: extracting cell type-specific information from heterogeneous samples. Curr Opin Immunol.

