# Supplementary material for: Integrated multi-cohort transcriptional meta-analysis of neurodegenerative diseases
Source: Acta Neuropathol Commun. 2014 Sep 4;2:93. doi: 10.1186/s40478-014-0093-y (PMC4167139; doi:10.1186/s40478-014-0093-y)
Supplement: Supplementary file 2 — Additional file 2: Figure S1.: CNM genes are consistently differentially expressed in samples in the discovery and validation data sets. Figure S2. CNM genes are consistently differentially expressed in samples in the secondary validation data sets. Figure S3. Complete annotations for Figure 4A. Figure S4. Forest plots for highlighted hub genes of interest. Figure S5. Network and pathway analyses reveal common pathways and hubs in neurodegeneration. Figure S6. Heat map of CNM genes and their correlation with aging in three independent aging brain data sets. (PDF 13 MB) [file 40478_2014_9093_MOESM2_ESM.pdf]

Supplementary Figures

INTEGRATED MULTI-COHORT TRANSCRIPTIONAL META-ANALYSIS OF NEURODEGENERATIVE DISEASE

Matthew D. Li*[1], Terry C. Burns*[1,2], Alexander A. Morgan[1], Purvesh J. Khatri[1,3]

* These authors contributed equally to this work

Affiliations:

[1] Stanford University School of Medicine
Stanford, CA 94305, USA

[2] Department of Neurosurgery
Stanford University
Stanford, CA 94305, USA

[3] Stanford Institute for Immunity, Transplantation and Infection (ITI)
Stanford Center for Biomedical Informatics Research (BMIR)
Department of Medicine
Stanford University
1265 Welch Rd., MSOB X219, Stanford, CA 94305, USA

Corresponding Author:

Purvesh J. Khatri, Ph.D.
Phone: 313-433-2836
Email: pkhatri@stanford.edu

SUPPLEMENTARY FIGURES – TABLE OF CONTENTS

Supplementary Figure 1. CNM genes are consistently differentially expressed in samples in the discovery and validation data sets.

Supplementary Figure 2. CNM genes are consistently differentially expressed in samples in the secondary validation data sets.

Supplementary Figure 3. Complete annotations for Figure 4A.

Supplementary Figure 4. Forest plots for highlighted hub genes of interest.

Supplementary Figure 5. Network and pathway analyses reveal common pathways and hubs in neurodegeneration.

Supplementary Figure 6. Heat map of CNM genes and their correlation with aging in three independent aging brain data sets.

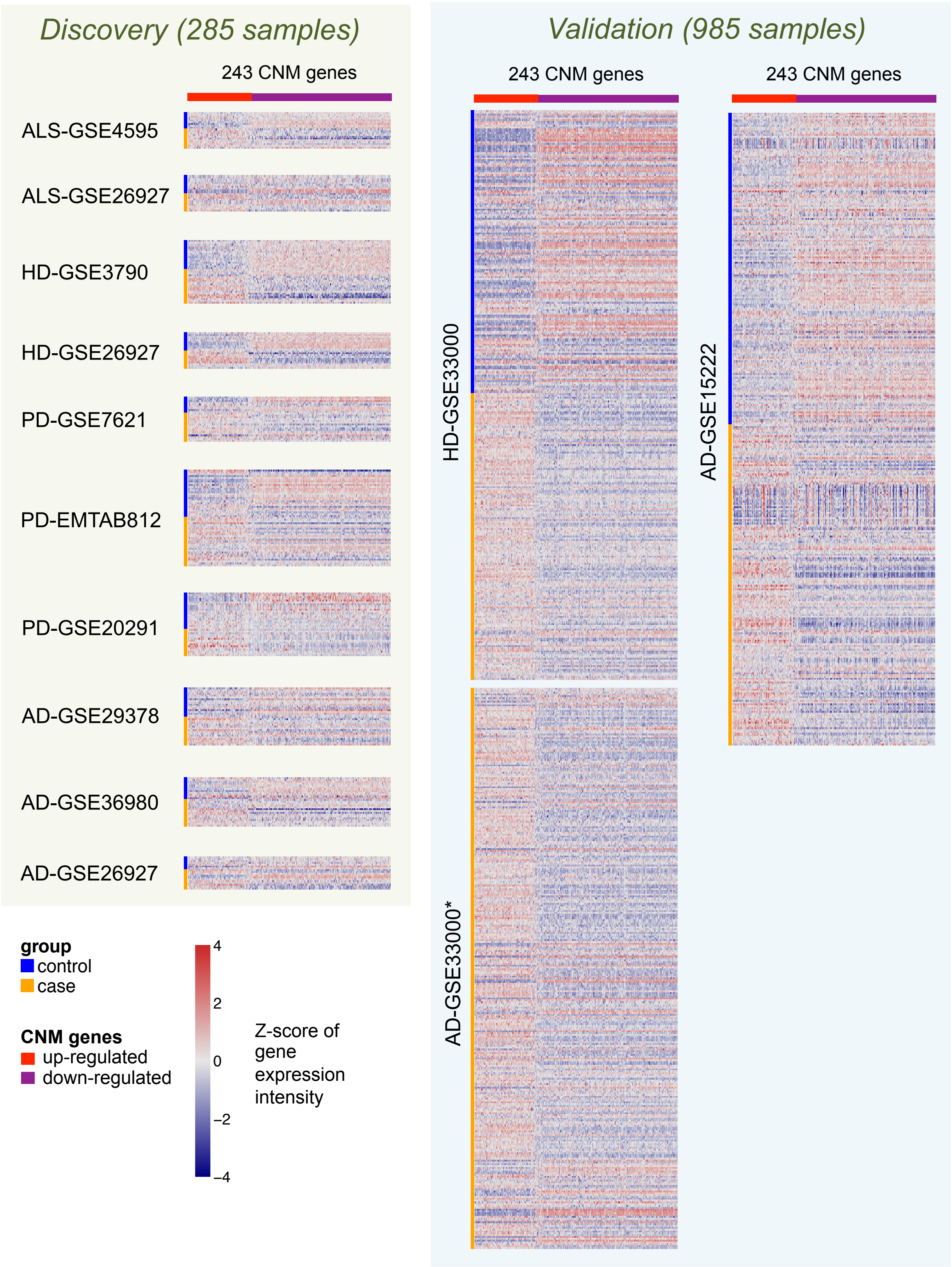

Supplementary Figure 1. CNM genes are consistently differentially expressed in samples in the discovery and validation data sets. Heat map rows denote samples, while columns correspond to the 243 CNM genes, ranked from left to right from highest to lowest log2 standardized mean difference (Hedges' *g*). Refer to Table 1 for data set information. ALS, amyotrophic lateral sclerosis; HD, Huntington's disease; PD, Parkinson's disease; AD, Alzheimer's disease. *AD-GSE33000 control samples are the same as HD-GSE33000 control samples.

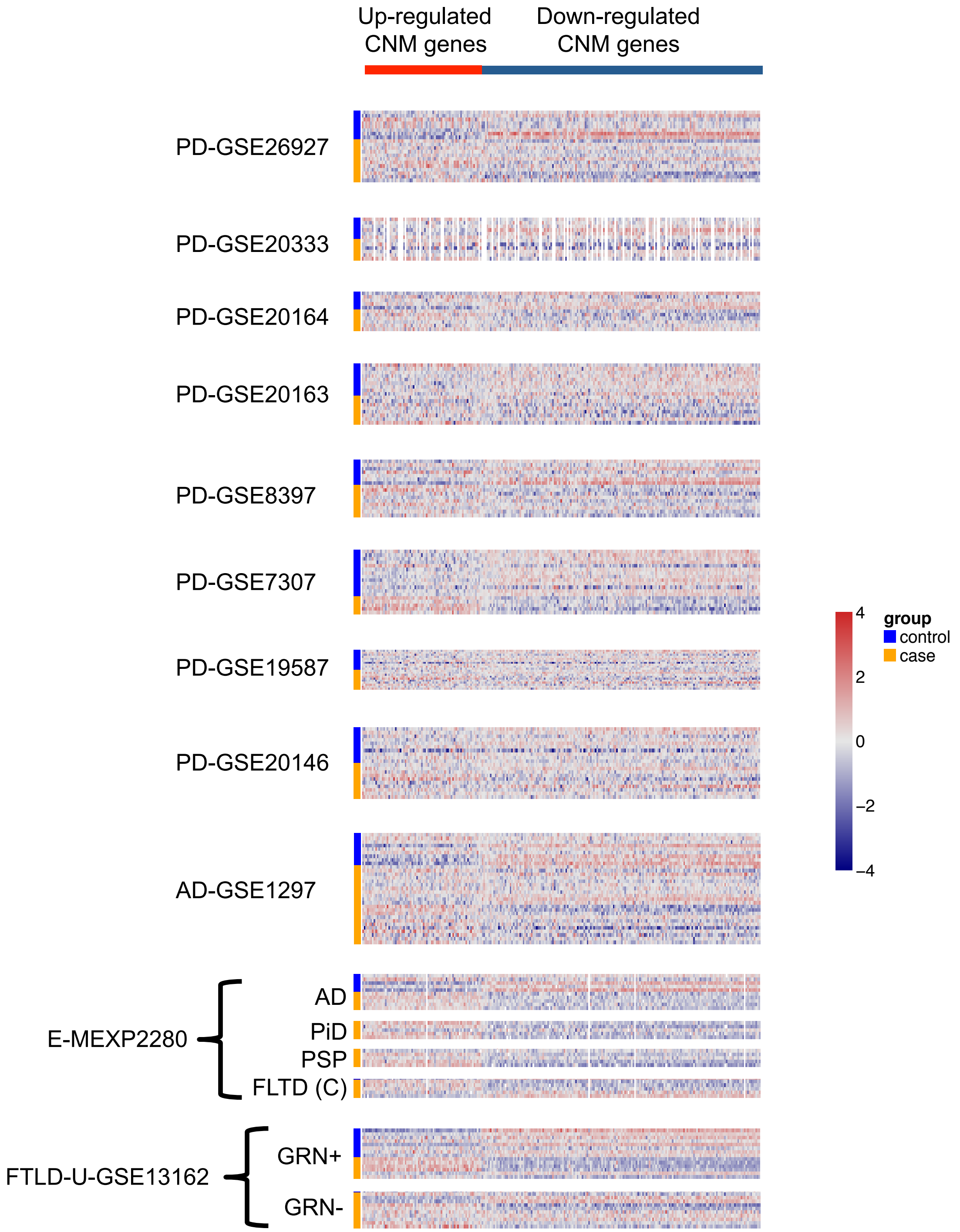

Supplementary Figure 2. CNM genes are consistently differentially expressed in samples in the secondary validation data sets. Heat map rows denote samples, while columns correspond to the 243 CNM genes, ranked from left to right from highest to lowest log2 standardized mean difference (Hedges' *g*). Refer to Table 1 for data set information. Note that Supplementary Figure 2 is proportionally enlarged relative to Supplementary Figure 1. PD, Parkinson's disease; AD, Alzheimer's disease; PiD, classical Pick's disease, FTLD, frontotemporal lobar dementia (Constantinidis type C); PSP, progressive supranuclear palsy; FTLD-U-GRNpos, frontotemporal lobar dementia with ubiquitin- and TDP-43-positive inclusions, progranulin mutation positive; FTLD-U-GRNneg, frontotemporal lobar dementia with ubiquitin- and TDP-43-positive inclusions, progranulin mutation negative.

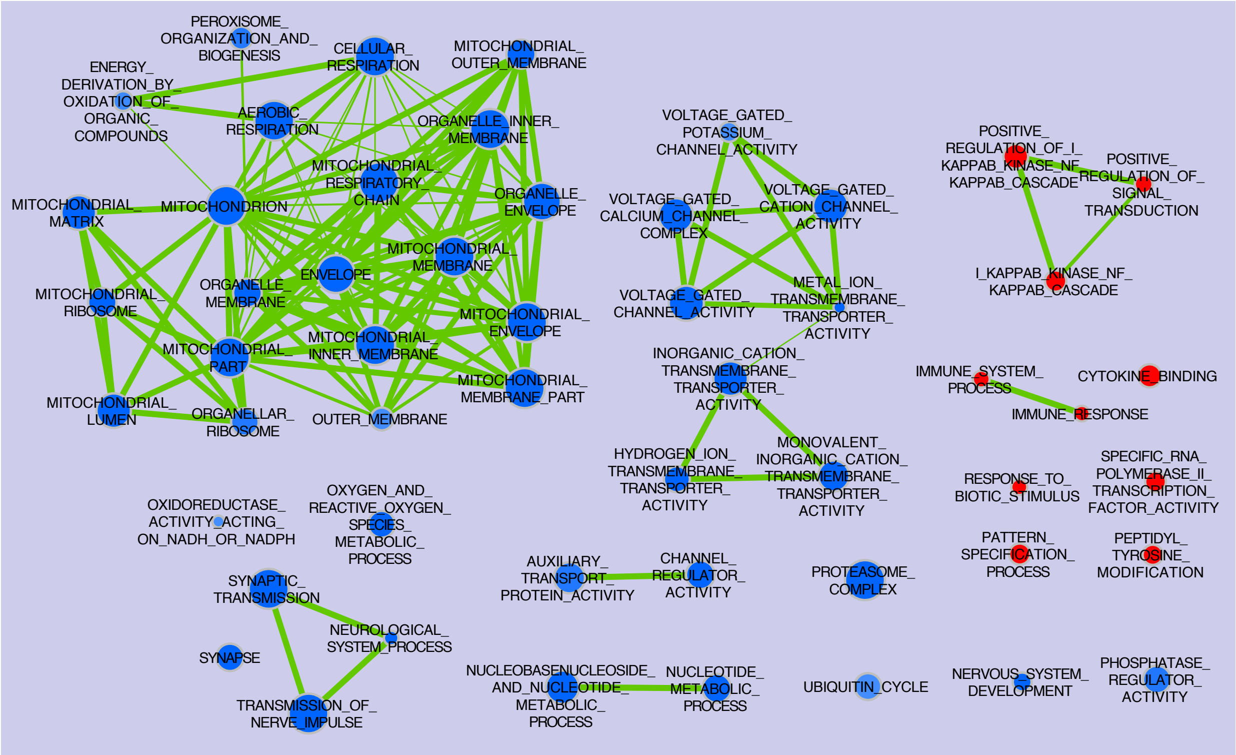

Supplementary Figure 3. Complete annotations for Figure 4A. EnrichmentMap [44] network for overlapping enriched Gene Ontology gene sets identified by GSEA. Each node represents a significantly enriched gene set (FDR q-value ≤ 0.05), and more significant nodes are proportionally larger. Red nodes denote gene sets enriched in neurodegenerative disease tissue, while blue nodes denote those enriched in control tissue. Green lines appear between any gene sets with > 50% overlap, and are proportionally thicker given greater overlap. See Supplementary Figure 3 for full annotations of nodes. (B-D) MetaCore analyses generated, inputting all 243 CNM genes.

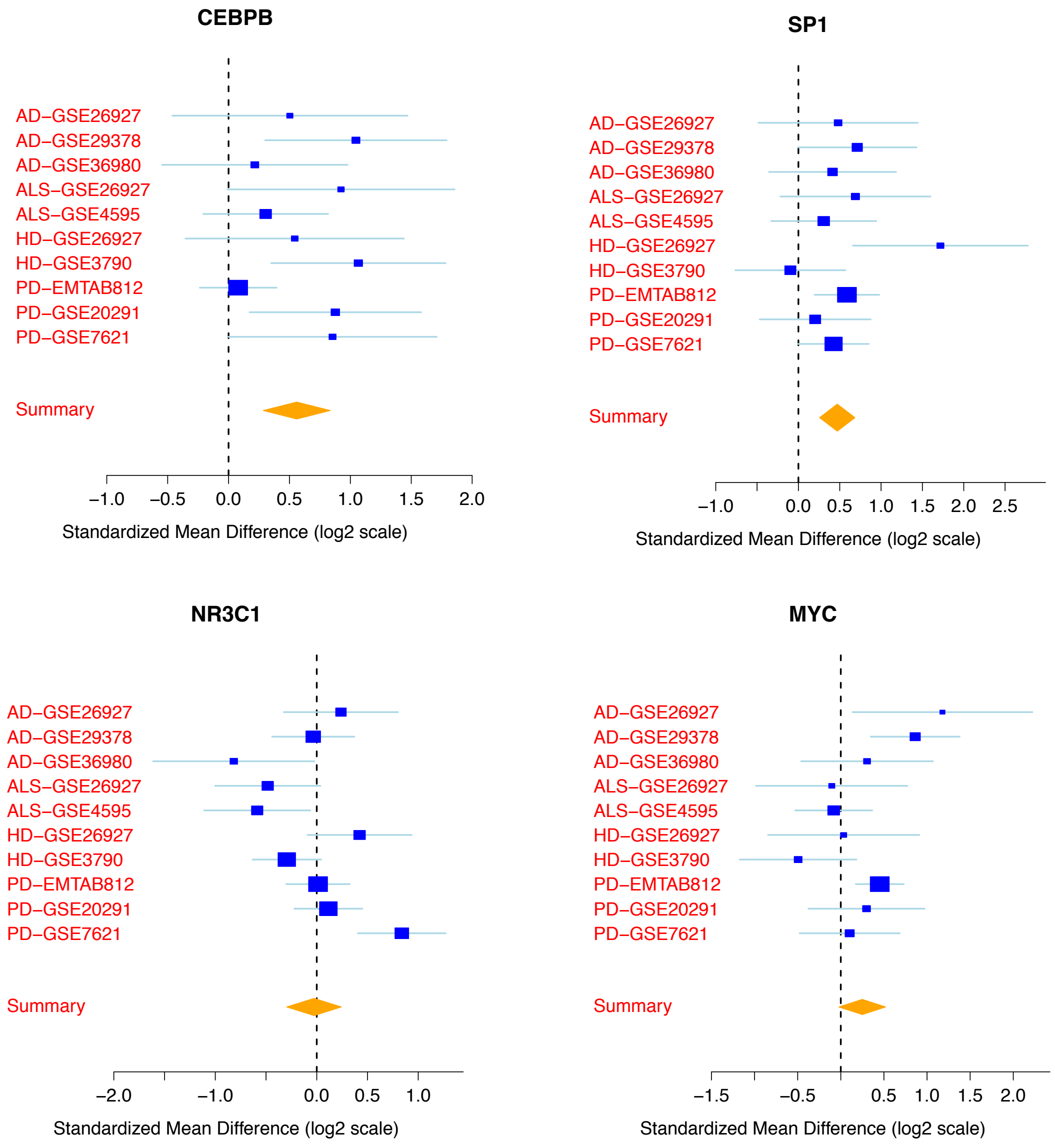

Supplementary Figure 4. Forest plots for highlighted hub genes of interest. Forest plot x-axes show standardized mean difference (Hedges' g in log2 scale) for genes in multiple data sets. Blue box sizes are inversely proportional to the SEM difference of the gene in each data set. Whiskers denote 95% confidence interval. Yellow diamonds represent combined mean difference for each gene. Yellow diamond width denotes 95% confidence interval.

A

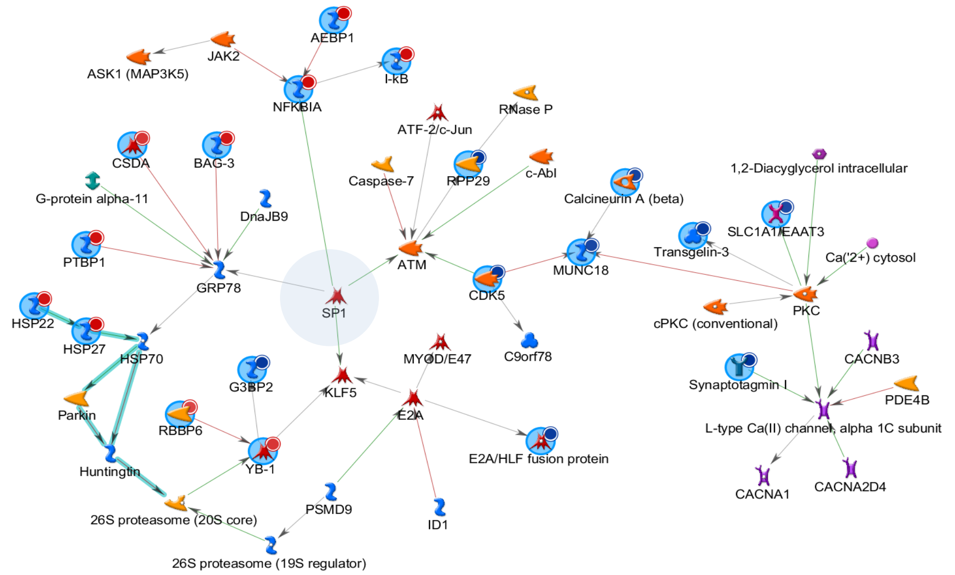

B

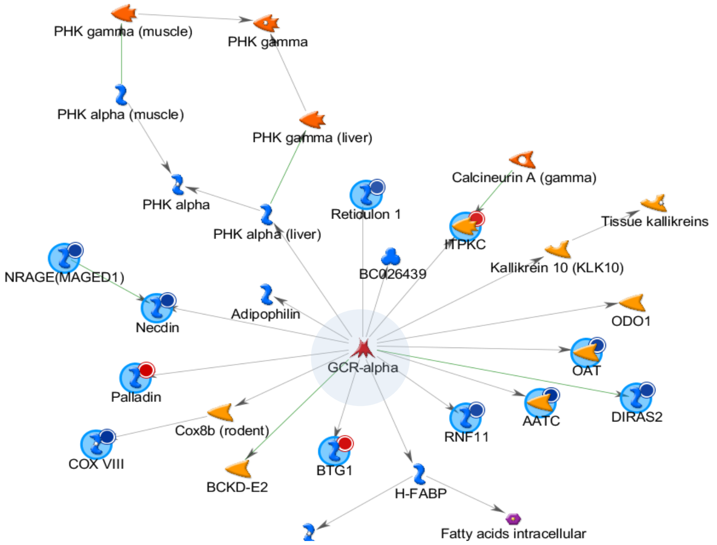

Supplementary Figure 5. Network and pathway analyses reveal common pathways and hubs in neurodegeneration. MetaCore analyses generated networks, inputting all 243 CNM genes. First (A), Second (B) and third (B) most significant network generated using the default network analysis. Symbols surrounded by blue circles are CNM genes. Smaller red and blue circles denote up-regulated and down-regulated genes respectively. Refer to MetaCore website for detailed network symbol legend.

C

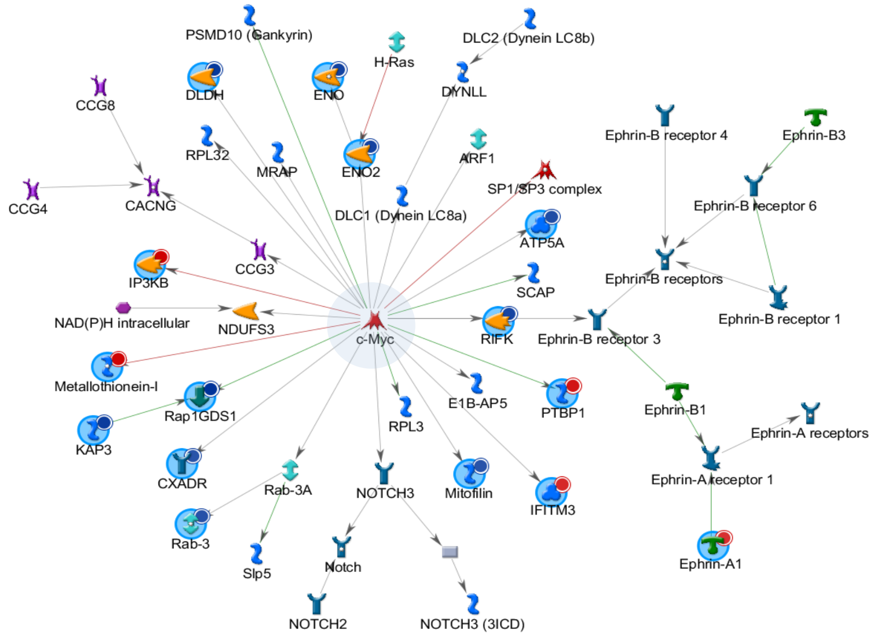

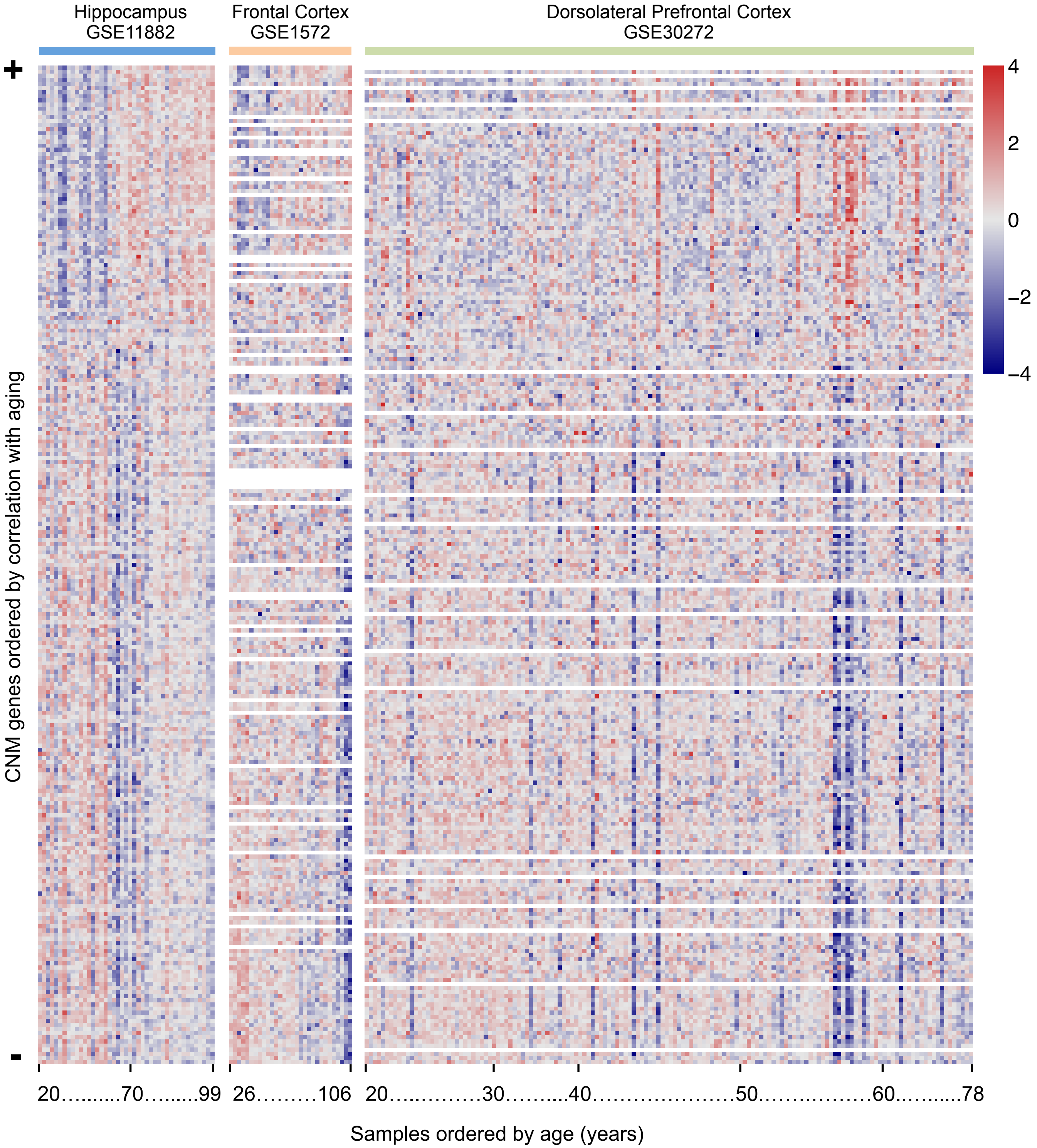

Supplementary Figure 6. Heat map of CNM genes and their correlation with aging in three independent aging brain data sets. Rows denote CNM genes ranked from highest to lowest mean correlation with aging, from top to bottom. Columns denote samples in normal aging brain data sets, ordered from young to old.
