# Supplementary figures and images for: Integrated multi-cohort transcriptional meta-analysis of neurodegenerative diseases
Source: Acta Neuropathol Commun. 2014 Sep 4;2:93. doi: 10.1186/s40478-014-0093-y (PMC4167139; doi:10.1186/s40478-014-0093-y)

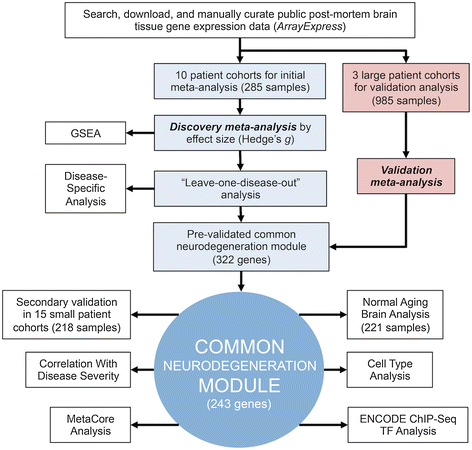

Supplement: Supplementary file 3 — Authors’ original file for figure 1 [file 40478_2014_9093_MOESM3_ESM.gif]

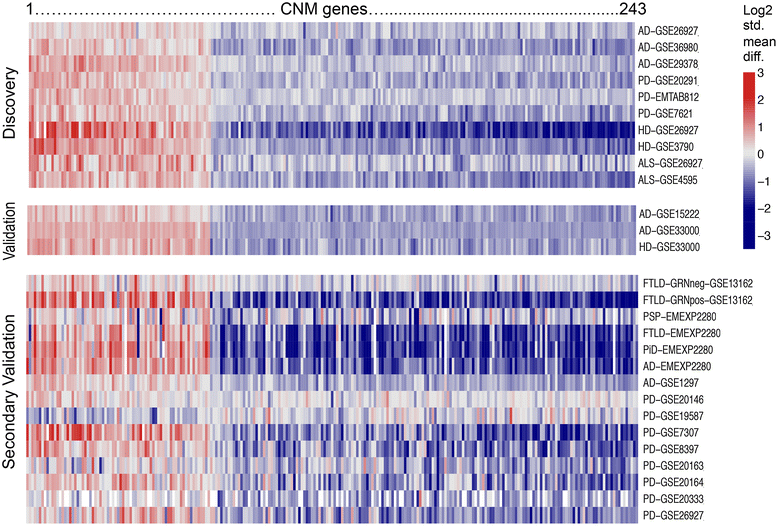

Supplement: Supplementary file 4 — Authors’ original file for figure 2 [file 40478_2014_9093_MOESM4_ESM.gif]

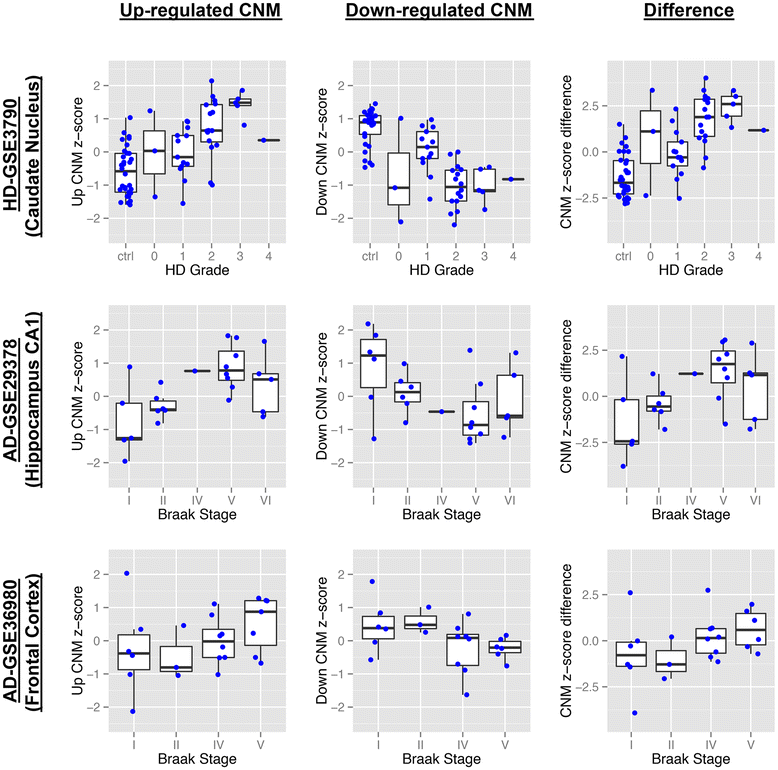

Supplement: Supplementary file 5 — Authors’ original file for figure 3 [file 40478_2014_9093_MOESM5_ESM.gif]

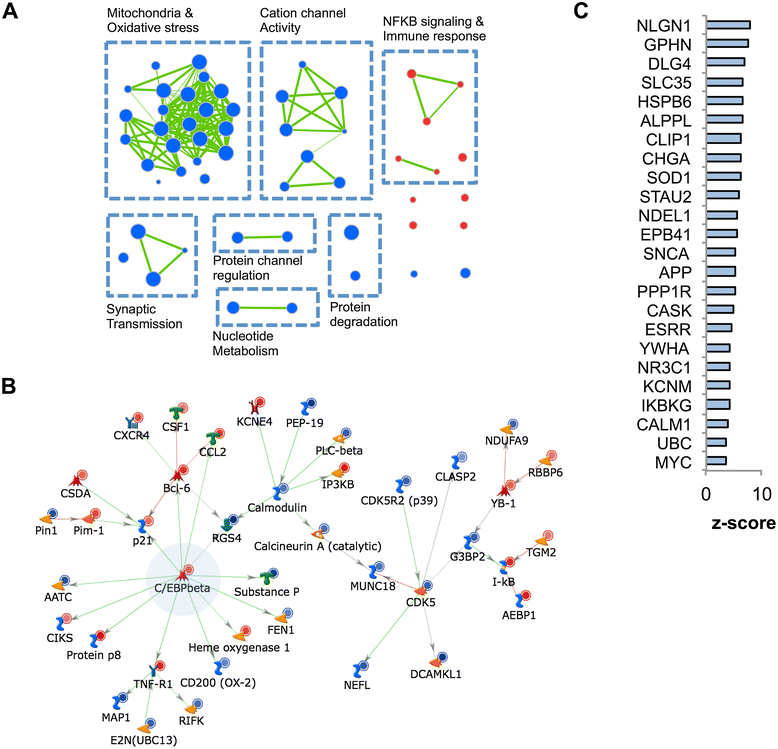

Supplement: Supplementary file 6 — Authors’ original file for figure 4 [file 40478_2014_9093_MOESM6_ESM.gif]

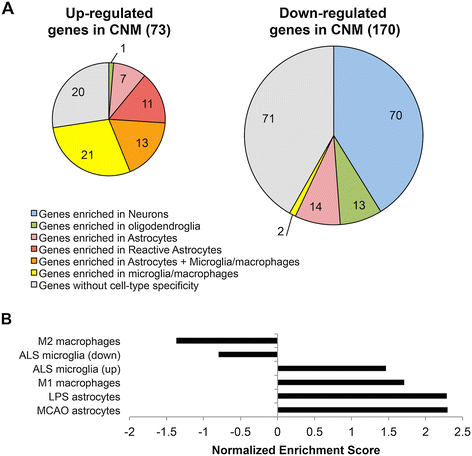

Supplement: Supplementary file 7 — Authors’ original file for figure 5 [file 40478_2014_9093_MOESM7_ESM.gif]

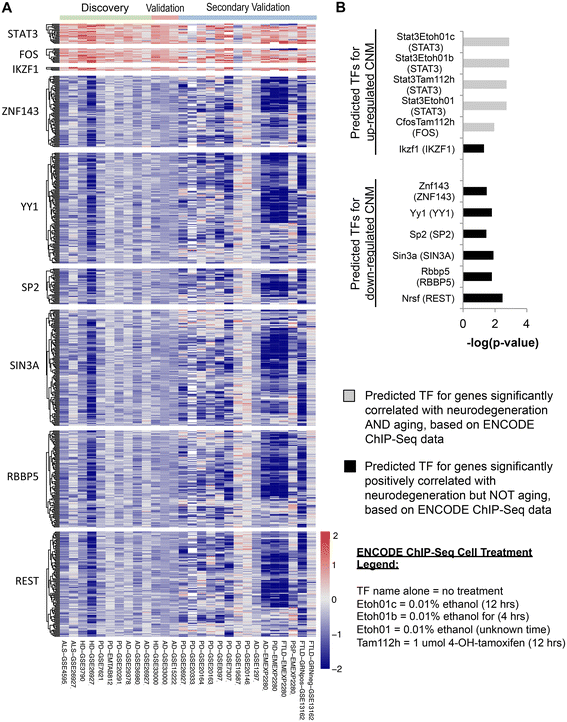

Supplement: Supplementary file 8 — Authors’ original file for figure 6 [file 40478_2014_9093_MOESM8_ESM.gif]

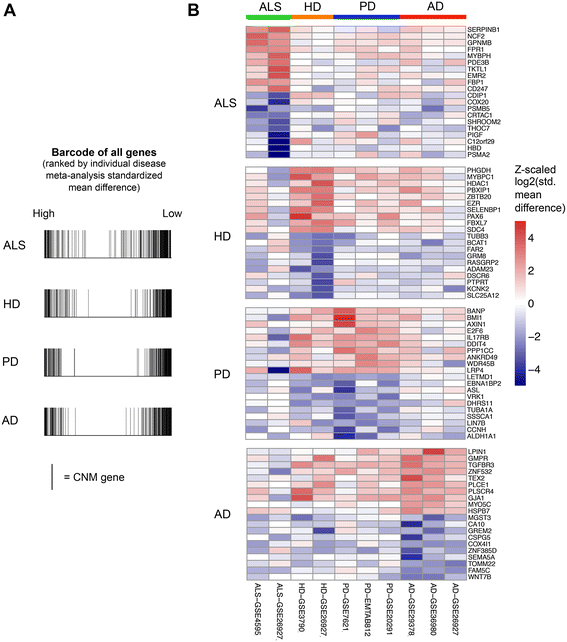

Supplement: Supplementary file 9 — Authors’ original file for figure 7 [file 40478_2014_9093_MOESM9_ESM.gif]

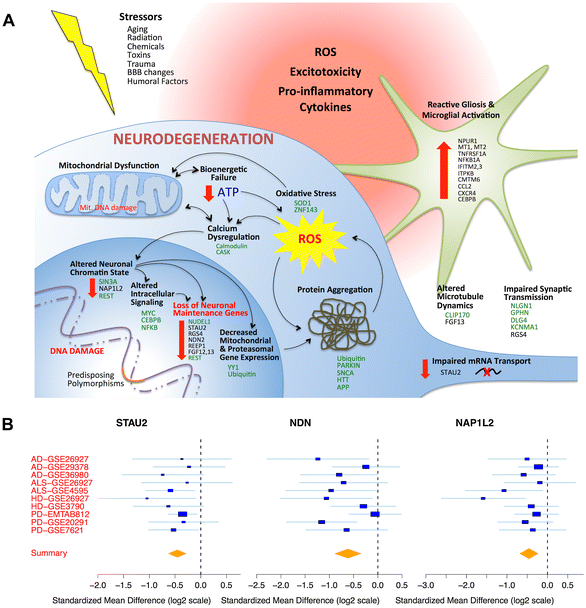

Supplement: Supplementary file 10 — Authors’ original file for figure 8 [file 40478_2014_9093_MOESM10_ESM.gif]
